# The human factor H protein family – an update

**DOI:** 10.3389/fimmu.2024.1135490

**Published:** 2024-02-12

**Authors:** Noémi Sándor, Andrea E. Schneider, Alexandra T. Matola, Veronika H. Barbai, Dániel Bencze, Hani Hashim Hammad, Alexandra Papp, Dorottya Kövesdi, Barbara Uzonyi, Mihály Józsi

**Affiliations:** ^1^ Department of Immunology, ELTE Eötvös Loránd University, Budapest, Hungary; ^2^ HUN-REN-ELTE Complement Research Group, Hungarian Research Network, Budapest, Hungary

**Keywords:** Factor H, factor H-related proteins, complement alternative pathway, infection, inflammation, cancer, age-related macular degeneration, kidney disease

## Abstract

Complement is an ancient and complex network of the immune system and, as such, it plays vital physiological roles, but it is also involved in numerous pathological processes. The proper regulation of the complement system is important to allow its sufficient and targeted activity without deleterious side-effects. Factor H is a major complement regulator, and together with its splice variant factor H-like protein 1 and the five human factor H-related (FHR) proteins, they have been linked to various diseases. The role of factor H in inhibiting complement activation is well studied, but the function of the FHRs is less characterized. Current evidence supports the main role of the FHRs as enhancers of complement activation and opsonization, i.e., counter-balancing the inhibitory effect of factor H. FHRs emerge as soluble pattern recognition molecules and positive regulators of the complement system. In addition, factor H and some of the FHR proteins were shown to modulate the activity of immune cells, a non-canonical function outside the complement cascade. Recent efforts have intensified to study factor H and the FHRs and develop new tools for the distinction, quantification and functional characterization of members of this protein family. Here, we provide an update and overview on the versatile roles of factor H family proteins, what we know about their biological functions in healthy conditions and in diseases.

## Introduction

Complement is an ancient and complex network of the immune system and is interconnected with other protein networks, such as the coagulation system and the kinin system. In addition to its role in protection against infections, the complement system plays pivotal roles in regulating waste disposal, such as the removal of immune complexes, cell debris, dying cells, and amyloids, as well as the elimination of synapses in the central nervous system. Complement is important in the regulation of inflammation, and of the function and activity of various immune and non-immune cells. Disturbance in these physiological functions may lead or contribute to diseases, therefore the system is tightly regulated and controlled (reviewed in 1–3).

**Figure 1:**
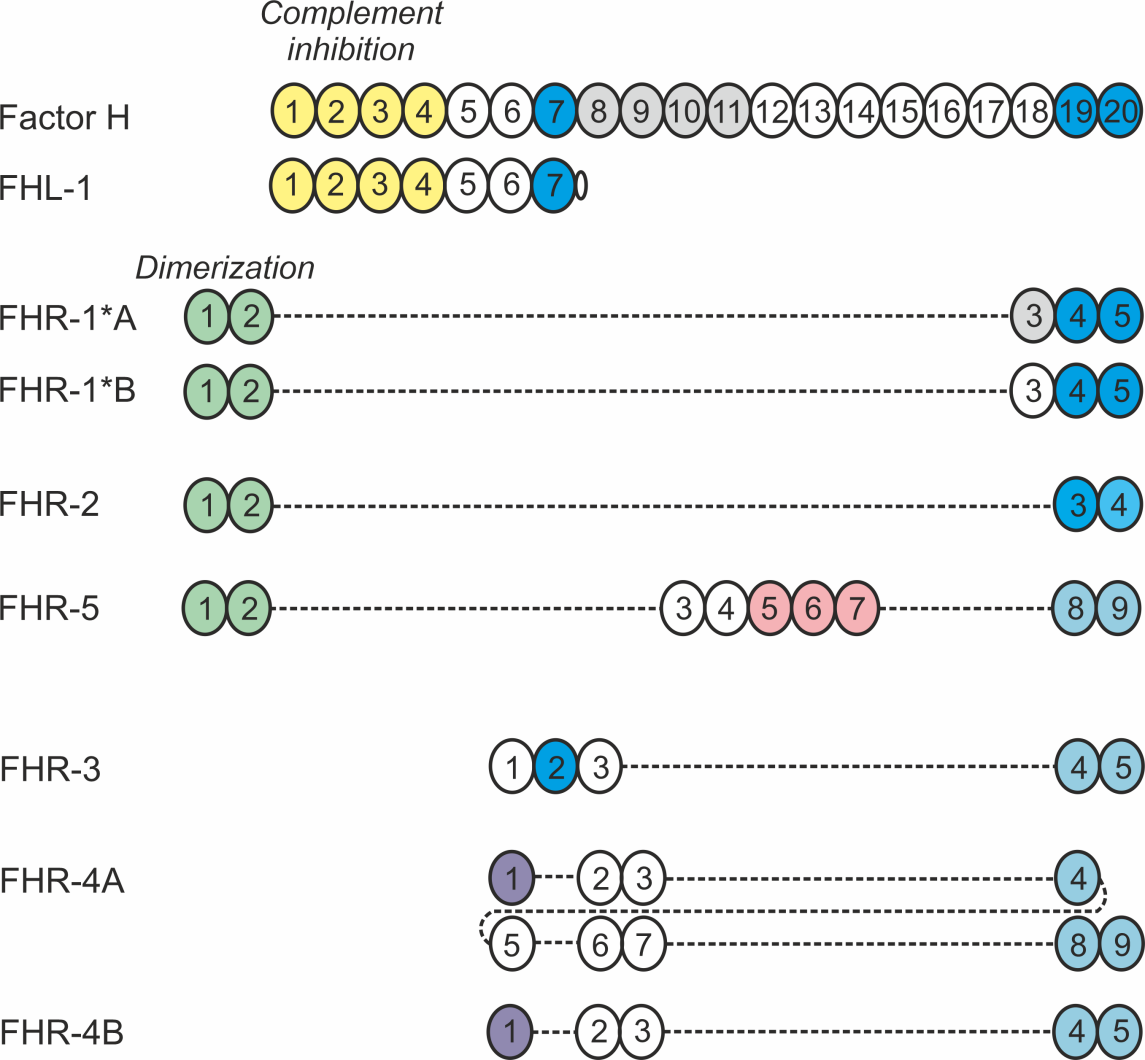
Schematic representation of the human FH protein family. Members of the human FH protein family are exclusively composed of 4 to 20 CCP domains, represented as circles. The numbers indicate the individual domains, and coloring indicates domains responsible for binding to ligands and/or mediating functions. Domains of the FHR proteins display varying degree of amino acid sequence identity to the corresponding FH domains and are vertically aligned accordingly (5). Please note that the existence of FHR-4B is controversial (6). Figure modified after 7.

Factor H (FH) is one of the complement regulatory proteins and is involved in most of the above-mentioned complement functions. Its major role is the downregulation and inhibition of the alternative complement pathway and the amplification loop (4). FH, its splice variant FHL-1 and five factor H-related (FHR) proteins that are transcribed from five separate genes in direct vicinity of the *CFH* gene, constitute the FH protein family (reviewed in (5) (**Figure 1**). These soluble proteins are all exclusively composed of complement control protein (CCP) domains. FH is built up of 20 CCPs, of which the N-terminal domains mediate the complement inhibiting functions of FH, whereas CCPs 6-7 and 19-20 are involved in binding to various ligands in fluid phase or on surfaces (**Figure 1**). FHL-1 is a shorter form containing essentially the N-terminal 7 CCPs, whereas the FHRs lack CCPs homologous to the complement regulatory domains (CCPs 1-4) but retained domains that are homologs of the ligand-binding domains of FH (5, 8).

All FH family proteins interact with the C3b fragment of the central complement component, C3. FH and FHL-1 have cofactor activity, as they support C3b cleavage by factor I; they also compete with factor B for C3b binding, thus preventing the formation of the C3bBb convertase, and have decay accelerating activity by facilitating the disassembly of the alternative pathway C3 convertase and the C5 convertases. The FHR proteins have negligible such activities, as explained by the lack of the FH CCP1-4 homolog domains. Instead, some of the FHRs were shown to recruit C3b to surfaces where they are bound and support C3 convertase formation and thus complement activation. In addition, they can compete with FH for binding to C3b, as well as to certain other ligands (5). Nevertheless, while several studies attest to the role of both FH and FHRs in disease, the functions of the latter are still poorly understood. This review summarizes what we know and what we do not know about these proteins and puts these information and controversies into perspective.

## Ligands and functions of FH family proteins

**Figure 2:**
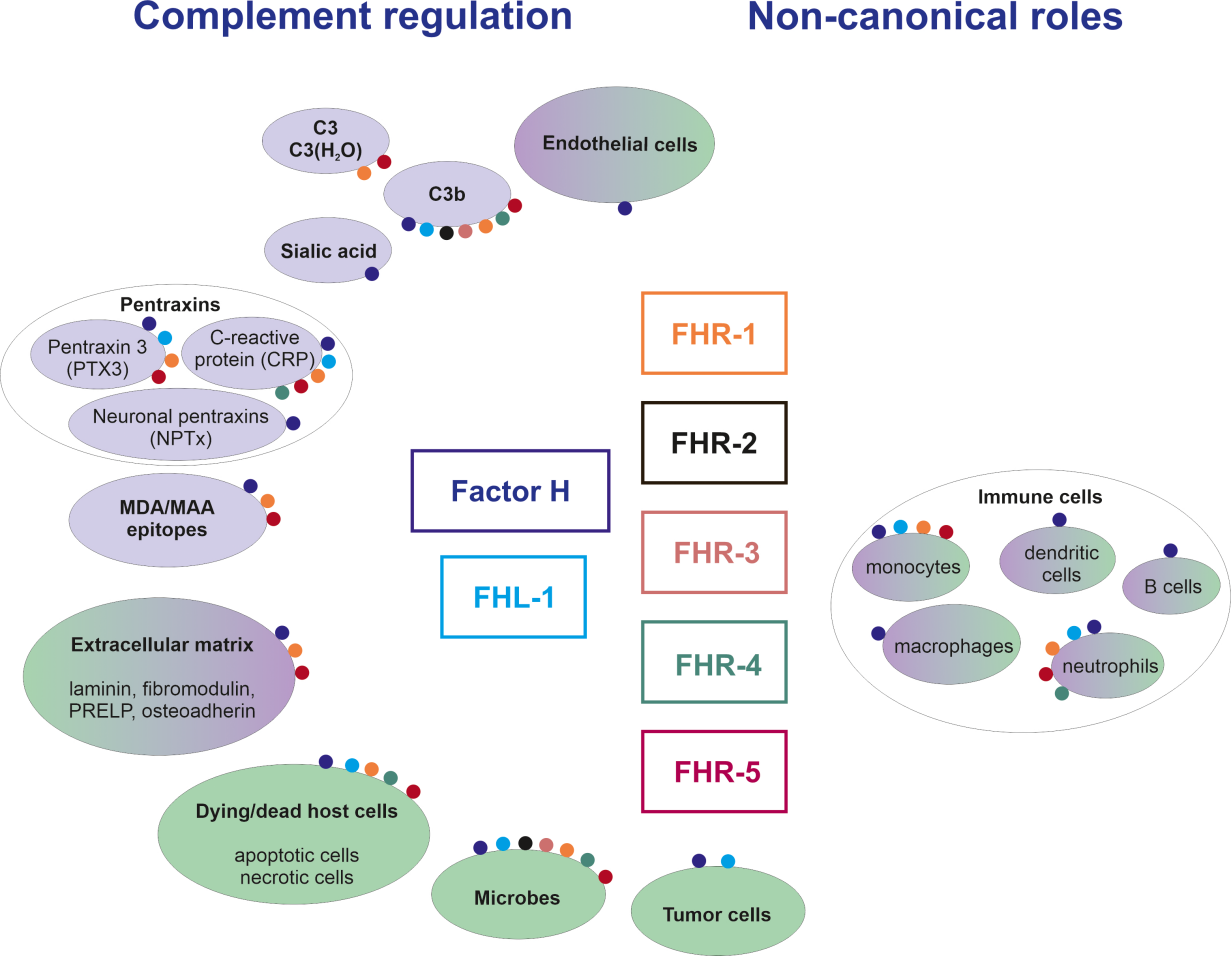
Overview of ligands and functions of the FH family proteins. The figure shows major ligands (in purple background) and cells/surfaces (in green background) to which binding of individual FH family members was shown. The interactions are represented by colored dots; the different colors code for the various FH family proteins as indicated in the middle of the figure. Note that non-canonical roles include effects also on non-immune cells, as discussed in the text.

**Figure 3:**
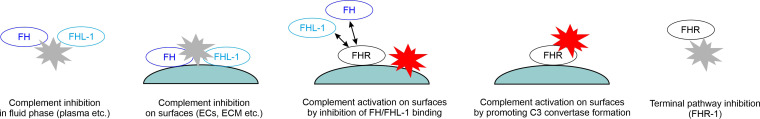
Roles of FH family proteins in the regulation of complement activation. The schematic drawing indicates the main funtions of FH and FHL-1 in inhibiting complement activation in body fluids and also when bound to certain surfaces, such as endothelial cells (ECs) and extracellular matrices (ECM; e.g. the glomerular basement membrane and the Bruch’s membrane). FHR proteins in turn may compete with the binding of FH (and FHL-1) to ligands and surfaces, thereby indirectly enhancing complement activation, but may also directly activate complement when bound to surfaces and – by binding C3, C3(H_2_O) or C3b – recruiting a functional C3 convertase. In addition, some FHRs were reported to inhibit C5 conversion and the terminal pathway.

FH was discovered more than 50 years ago (9), and its structure and function have been extensively studied since then (10). Numerous FH ligands have been identified and several of these ligands were also studied for their potential interaction with FHRs. The detailed description of these is beyond the scope of this manuscript, and we refer the readers to other excellent reviews on this topic (7, 11). Instead, here we focus on major ligands only, also in the light of new data, and attempt to synthesize this knowledge, reconcile controversies and interpret them considering proposed and possible functions (**Figures 2**, **3**).

The main role of FH and FHL-1 is the downregulation of complement activation, which requires their interaction with C3b via their N-terminal CCPs 1-4. In addition, FH binds to surface deposited C3b and C3d through CCPs 19-20. Because of the sequence similarities of FHRs to FH, they all bind the main FH ligand C3b, and/or its cleavage products iC3b and C3d. FHRs were reported to bind C3b via their C-terminal domains (12–15), and FHR-5 has an additional C3b binding site in its middle region (16). FHR-3 and FHR-4 have no detectable cofactor activity at physiological concentrations, rather they somewhat enhanced the cofactor activity of FH, an activity detected at supraphysiological concentrations only (17). Inhibition of fluid-phase alternative pathway C3 convertase was reported for FHR-2 (14) and FHR-5 (18); and cofactor activity for FHR-3 (19) and FHR-5 (18) was reported, although these are likely not relevant under physiological conditions as they were detected using high FHR concentrations.

In contrast to FH, C3b binding by FHRs enhances the activation of the alternative pathway by allowing formation of the alternative pathway C3 convertase. Surface-bound FHR-1, FHR-4 and FHR-5 increased the deposition of C3-fragments, the factor B fragment Bb, and properdin from serum and an active C3 convertase could also be assembled on them using purified proteins (13, 20, 21). In addition, FHR-1 was recently reported to bind native C3 directly, thus promoting local complement activation (22, 23). Moreover, FHR-5 was shown to bind native C3 and C3(H_2_O) (16). Since native C3 and C3 with hydrolized thioester bond, C3(H_2_O), are constantly available, while C3b generation requires prior complement activation, the direct C3 and/or C3(H_2_O) binding may represent the major mechanism by which FHR-1 and FHR-5 enhance alternative pathway activation. Thus, similar to properdin, FHRs emerge as soluble pattern recognition molecules that bind to certain surfaces/surface ligands and there they locally promote alternative pathway activation and opsonization. FHR-1 and FHR-5 enhanced alternative pathway activation on different surfaces, such as pentraxins, extracellular matrix (ECM), apoptotic and necrotic cell-surfaces, and genomic DNA as well (15, 16, 20, 21, 24, 25).

The observed complement activation enhancing effect of FHRs may be the combined outcome of direct activation of the alternative pathway and the competitive inhibition of FH binding to these ligands and surfaces. FHR proteins can compete with FH for binding to surface bound C3b, thus decreasing the inhibitory activity of FH, a process termed FH deregulation (23, 26, 27). Additionally, FHRs compete with FH for binding to other ligands, including pentraxins and ECM, as it was shown previously in the case of FHR-5, which dose-dependently inhibited the binding of FH to monomeric C-reactive protein (mCRP), pentraxin 3 (PTX3), and MaxGel, the latter used as an *in vitro* model for ECM, resulting in reduced cofactor activity of FH (20, 21). FHR-5 binds to mCRP, PTX3 and ECM proteins such as laminin, fibromodulin, osteoadherin and proline/arginine-rich end leucine-rich repeat protein via CCPs 3-7 (15, 16, 18, 25). Pentraxins, like CRP and PTX3 are soluble pattern recognition molecules which bind to pathogens, apoptotic cells, and ECM components and at the same time interact with different complement proteins, including cascade activators such as C1q and MBL, as well as FHR-1 and FHR-5, and inhibitors like FH and C4BP. These interactions on host ligands allow opsonization of target surfaces without initiating excessive complement activation and terminal pathway activation (28). Surface-bound pentraxins recruit FH to apoptotic cells which reduces complement activation and inhibits inflammatory processes. FH binds to apoptotic cells via Annexin-II, DNA, histones (29), and malondialdehyde epitopes as well (30). Next to FH, FHR-5 binds to malondialdehyde epitopes and malondialdehyde-acetaldehyde adducts via CCPs5-7 and activates complement (15). FHR-1 and FHR-3 were also shown to compete with FH for binding to MDA epitopes, and FHR-1 was suggested to inhibit FH activity on MDA epitopes (31). FHR-1 and FHR-5 were reported to bind to dead cells (necrotic and apoptotic) presumably through DNA, based on binding assays and colocalization data (25). FH interacts with DNA via CCPs 6-9 and CCPs 19-20, while FHR-5 binds to DNA through its CCPs 3-7 and competes with FH, therefore reduces the cofactor activity of FH (25).

Furthermore, FH binds to sialic acid via CCP20, and glycosaminoglycans (GAGs) and heparin via CCP7 and CCP20 (32–35), thereby differentiates self and non-self surfaces to prevent complement-mediated injury of the host (36–39). FHR-3 has a heparin-binding site in CCP2 (17), while FHR-5 binds to heparin via CCPs 5-7 (18). The interaction of FHR-5 with sulfated GAGs was recently studied in detail and it was found that in the presence of sulfated GAG on the surface, FHR-5 can more strongly reduce FH binding to surface-bound C3b than without GAG present (40). FHR-1 was also reported to bind to heparin and cellular surfaces and deregulates FH (12, 41). The aHUS-associated FHR-1 variant with amino acid substitutions L290S and A296V (42) gains sialic acid binding, which results in an increased capacity of this mutant FHR-1 to deregulate complement (43).

FH, FHL-1, and FHRs can also interact with non-cellular surfaces, like the ECM. Components of the ECM may be exposed to body fluids due to e.g., tissue injury, cell activation or damage. Moreover, unique layers due to their specialized anatomy are more exposed to components of the blood, such as the Bruch’s membrane in the eye and the glomerular basement membrane in the kidney. On these layers FH and/or FHL-1 represent the main complement inhibitors, due to the lack of membrane-bound regulators. FH binds to non-cellular surfaces via CCPs 19-20 and FHL-1 binds through CCP7 in the Bruch’s membrane (44, 45). Interaction of FH and FHRs, as it was shown for FHR-1 and FHR-5, with the ECM is important especially in complement-mediated eye and renal diseases, discussed in detail later. All these recent data suggest that FHR proteins are positive regulators of the complement system and support complement activation mainly via the alternative pathway.

In view of the above-mentioned results, it may appear counterintuitive that FHR-1, FHR-2, and FHR-5 were reported to inhibit the terminal pathway, thus they are (also) complement inhibitors (12, 14, 46). However, these proteins may have a dual effect on complement, enhancing the early steps of activation and thus promoting opsonization, but at the same time inhibiting the inflammatory and the lytic terminal pathway. This may ensure a targeted but restricted complement activation optimized for the safe handling of altered self-cells and ligands (28, 47). Inhibition of C5 conversion could be due to the binding of the FHRs to densely deposited C3b, generated by the C3 convertases, thereby preventing the priming of C5 (46, 48).

It is important to note that the plasma concentrations of the FHRs are much lower than that of FH. There is considerable discrepancy in the literature regarding the absolute values (discussed in detail in 49). In general, for FH plasma concentrations between 1-2 µM (ca. 150-300 µg/ml) are reported, whereas recently reported concentrations for the FHRs, using well-characterized monoclonal antibodies, are well below 1 µM. For FHR-1 0.35 µM (10-15 µg/ml) was measured, and even less for the other FHRs; their combined concentration is ~0.5 µM (reviewed in 49). Since their action appears to be relevant on various surfaces, the affinities of the FHRs (and their disease-associated variants) towards the different ligands need to be measured, which relative to those of FH will determine the degree of complement deregulation locally. Also, the concentrations of the FH family proteins in other body fluids and in tissues, as well as under various pathological conditions, may markedly differ from those reported for blood plasma of healthy donors and need to be determined in future studies. Concentration determination as well as functional studies are further complicated by the homo- and heterodimerization of FHR-1, FHR-2 and FHR-5.

FH family proteins interact with altered self also in form of tumor cells and with invading microbes that hijack complement regulators to protect themselves from complement mediated damage and killing; FHRs may bind as countermeasures deployed by the host to defuse this complement evasion strategy (50–52), as discussed later in the present review.

In addition to their role in complement regulation, FH family proteins can bind to receptors on cell surfaces, thereby modulating cell activation and cellular functions, such as inflammatory responses, phagocytosis, cytokine production, and cell migration (53), discussed later in this review in more detail.

## What we learned from diseases/disease associations

As discussed above, there are some controversies regarding the physiological roles of the FHRs. While their functions are not clearly defined yet, genetic studies and FHR protein quantitative measurements revealed correlations between FHRs and diseases, including complement mediated inflammatory diseases (particularly age-related macular degeneration (AMD) and several kidney diseases) and infections, lending support to their proposed complement modulatory role (49, 54). Here, we highlight the role of FH family proteins in selected diseases.

### FH family proteins in eye and kidney diseases

Genetic and molecular studies underline the role of the complement system in the pathogenesis of inflammatory eye and kidney diseases. Complement activation is strictly regulated in body fluids and on host surfaces by secreted and/or membrane-bound molecules (1, 55). Complement dysregulation due to mutations in complement proteins or complement targeting autoantibodies results in a variety of pathologies (2, 7, 56, 57). The chromosomal region containing the *CFH* and *CFHR* genes is prone to mutations and rearrangements. In the past two decades, numerous variations have been detected in various patient groups and connections with pathological conditions were revealed (**Supplementary Table 1**). Beside polymorphisms showing association with diseases and loss of function mutations of FH, rearrangements have also been reported that lead to missing or hybrid FHR proteins that result in altered plasma FH : FHR ratio or functional aberrations; in addition, function blocking pathogenic autoantibodies against FH were described (7, 50, 57–59).

AMD is the leading cause of vision loss over 60 years of age in economically developed countries, affecting 8.7% of the whole world population (60). It is a chronic autoinflammatory disease, with several risk factors: predisposing genetic variations in complement genes, ageing, lifestyle, and environmental triggers and, despite intensive efforts, there is still no appropriate therapy curing the disease (61, 62). C3 glomerulopathy (C3G) describes diseases with glomerular C3 deposition without the presence of Ig, the two main forms being dense deposit disease (DDD) and C3 glomerulonephritis (63–65). The Bruch’s membrane in the eye and the glomerular basement membrane in the kidney lack membrane-bound complement regulators, therefore they are vulnerable to complement activation and complement mediated damage due to dysfunction or deregulation of the soluble regulators FHL-1 and FH (66). Lipid-rich deposits in the retina (called drusen) and in the glomeruli, and oxidative stress causing inflammation are common factors (66–68).

Alterations in CCPs 1-7 affect both FH and its splice variant FHL-1. The common FH variant (Y402H) affecting CCP7 of both FH and FHL-1 is a susceptibility factor to AMD and influences the binding of FH/FHL-1 to CRP and heparin, and prevents FH from targeting cellular debris for clearance, thereby causing inflammation (69–75). This variant was also detected in a patient with AMD and DDD showing that the mutation affects complement regulation at anatomically distinct sites of the body (76). Loss of function mutations in the *CFH* gene lead to dysfunctional FH and FHL-1 proteins, thereby increasing non-regulated C3 convertase levels and consequently, overwhelming complement activation in AMD (45, 77, 78) and C3G patients (7, 58, 79, 80).

In AMD, the deletion of the *CFHR1* and *CFHR3* genes confer protection (81); the protective effect of the deficiency of FHR-1 and FHR-3 is explained by their lack of competition with FH (31). This concept is further supported by recent results showing that higher levels of FHR-1, FHR-2, FHR-3, and FHR-4 proteins are associated with advanced AMD (82–84). In contrast, C3G is associated with mutations and rearrangements in the *CFHR* genes that generate FHR-1, FHR-2 and FHR-5 molecules with duplicated dimerization domains or hybrid FHR proteins essentially containing duplicated dimerization domains, that form larger multimers. These gain-of-function mutant FHRs have increased avidity to ligands and surfaces and are thus more effective competitors of FH than their native counterparts, leading to increased complement activation and C3G (27, 85–87).

Alternative pathway dysregulation is also involved in the pathology of IgA nephropathy, a common kidney disease. In most patients (~ 90%) C3 deposition was found next to IgA in the mesangium. The disease is associated with alterations in the *CFH*, *CFHR1* and *CFHR3* genes and with some rare variants in the *CFHR5* gene (88–90). The deletion of the *CFHR3* and *CFHR1* genes is protective in IgA nephropathy presumably via the lack of FH competitors (FHR-1, FHR-3) on the surfaces, leading to more effective inhibition of complement activation by FH.

aHUS is a thrombotic microangiopathy characterized by acute renal failure and is associated with alterations in complement genes (91). FH mutations may cause partial deficiency and in aHUS cluster in the C-terminus of the protein which is responsible for surface recognition (reviewed in 7). Two *CFHR* gene variants, the CFHR1*B and CFHR3*B variants also associate with aHUS (92, 93). In addition, gene conversion between *CFH* and *CFHR1* can result in mutant FH and FHR-1 proteins, and gene rearrangements give rise to FH::FHR-3, FH::FHR-1, FHR-1::FH hybrid proteins; all of which compete with the binding of wild type FH to cell surfaces and thus disturb complement regulation in aHUS (22, 42, 43, 59, 94, 95).

Despite the large number of variants, not all of them affect the function of FH (96). While several N-terminal FH mutations unfavorably affect FH functions (97, 98), a large cohort study of aHUS patients concluded that most FH mutations located in the N terminus or the middle region of the protein are not pathogenic (99). Recently, six rare genetic variants were described in aHUS, C3G and AMD patients altering the CCP1-4 of FH, but only one of them (Q81P) influenced the complement regulatory functions of the molecule significantly, and two other variants had minor effects (100). In contrast to this, in aHUS the proportion of pathogenic variants affecting the C terminus of FH was higher, pointing to the importance of the C-terminal domains (which is mainly responsible for surface binding) in complement regulation on surfaces in aHUS (101, 102). A special case is the C-terminal FH mutation R1210C that results in the covalent binding of FH to albumin, which prevents the interaction of FH with its natural ligands, and is associated with aHUS, C3G and AMD as well (102–105).

In aHUS and C3G not only *CFH* and *CFHR* variations, but autoantibodies against FH can also be present. FH autoantibodies in aHUS are often associated with deletion of the *CFHR3* and *CFHR1* or the *CFHR1* and *CFHR4* genes; it is in fact the lack of the FHR-1 protein which predisposes to the development of aHUS-associated FH autoantibodies (106–113). In aHUS, autoantibodies mainly bind to the C-terminus of FH; CCPs 19-20 of FH are important in self-surface recognition and ligand binding (e.g., C3b), thus autoantibodies can impair these C-terminal functions of the protein (108, 114, 115). In contrast to these, most FH autoantibodies in C3G bind to the N-terminal part of FH, reflecting the underlying differences between the pathomechanisms of aHUS and C3G (113, 116, 117). Altogether, autoantibodies can alter FH binding to ligands, impairing its complement regulatory activity in fluid phase and/or on surfaces, thus contributing to complement mediated pathologies. However, not all anti-FH antibodies are pathogenic or neutral, even in diseases affecting the kidney. In systemic lupus erythematosus, autoantibodies against FH were described that contributed to the lower prevalence of acute kidney injury, enhanced FH binding to C3b and improved its cofactor activity (118), thereby played a protective role.

### Factor H family proteins in neurological disorders

Alzheimer’s Disease (AD) is the most common cause of dementia affecting the elderly. Although it is subject of intensive research, the pathomechanism and the processes leading to AD are still unclear and biomarkers to predict the disease and its course are still not available. Several studies implicated the complement system and identified altered complement component levels in connection with AD. Here, we focus on the FH family proteins; for more detailed review on complement and AD, see 119–121.

Physiologically, complement components are synthesized in various cells of the brain but they can also get there from the plasma due to damage of the blood brain barrier. Increased FH and FHL-1 levels were detected in lysates from the brain tissue of AD patients by Western blot (122) and complement deposits including FH were detected histologically (123). By contrast, decreased level of FH was measured in stem-cell derived neurons from sporadic AD patients (124). Serum/plasma or cerebrospinal fluid are more easily accessible materials and several groups investigated FH levels in these body fluids. Data of these studies are controversial, elevated (125, 126), unchanged (127) and decreased (128, 129) FH levels were described. More consensus can be found on the use of FH plasma level alone or in combination with levels of other proteins to differentiate among neurodegenerative diseases or AD stages (126, 128–130). Polymorphisms of FH (and other complement genes) were analyzed in AD and an association of the 1277C allele (coding for the H402 polymorphic FH variant) with AD was found; however, the genetic model was different from that observed in AMD (131, 132).

Whether complement regulatory or non-canonical functions of FH are relevant in this setting is not clear. For example, FH can colocalize with heparan sulfate proteoglycans (agrin) in amyloid-β plaques and with complement receptor type 3 (CR3) in the AD brain. Thus, it was proposed that FH may promote the recognition of amyloid-β plaques by microglia through CR3 (122). CR3 is an opsonophagocytic receptor for complement-coated antigens on macrophages and several other cell types; in addition to iC3b it binds numerous ligands, including FH, and has versatile functions (133).

Elevated serum/plasma FH levels were measured in patients with two neurodegenerative, demyelinating inflammatory diseases, neuromyelitis optica spectrum disorder (NMOSD) and multiple sclerosis (134–136). In addition, autoantibodies against FH were described and shown to interfere with the FH-C3b interaction, thus possibly influencing complement regulation in some NMOSD patients (137).

Elevated FH and FHR-1,-2,-5 (measured together with a non-specific Ab recognizing all three FHRs) levels were detected in cerebrospinal fluid in NMOSD, clinically isolated syndrome and multiple sclerosis *versus* controls (138). It is not clear if these reflect the pathology of these diseases and/or represent a protective response of the body to these neuroinflammatory diseases. Since FH prevents excessive complement activation but FHRs may support opsonization, they may participate in the removal of debris and pathological molecules and damaged cells. Moreover, FHR-1 as terminal pathway inhibitor was proposed to have a beneficial effect, since FHR-1-expressing neural stem cells ameliorated brain injury in a mouse model of NMOSD (139). In addition, these FH family proteins may exert non-canonical roles outside complement regulation and help e.g., phagocytic activity of cells (53).

Further studies reported significantly increased FH and terminal complement complex in epilepsy (140), and increased plasma complement FH was found associated with geriatric depression (141). While these and the previously mentioned data strongly implicate complement and FH family proteins in neurological disorders, further studies are required to better understand the pathomechanisms of these diseases and the role of FH family proteins in them.

### FH and FHRs in oncological disorders

Increased expression of FH and FHL-1 by tumor cells as a protective measure against complement-mediated damage and killing – in addition to the upregulation of cell membrane-anchored complement inhibitory proteins – is known for long (142–145). The role of complement in tumors is complex and context-dependent (146, 147). An increasing body of evidence implicates FH family proteins in oncological diseases as briefly summarized here.

FH is expressed at higher levels in CNS lymphoma, and elevated FH levels were found in bronchoalveolar lavage and sputum of lung cancer patients (148–150); moreover, non-small cell lung cancer cells (NSCLC) and cell lines were shown to express and secrete FH and FHL-1 (151–153). Daugan et al. investigated the effects of membranous and intracellular FH in clear cell renal cell carcinoma and lung cancer (154). While membranous FH in its canonical role inhibited complement activation, it had no effect on tumor cell phenotype or patient survival. However, a non-canonical protumoral effect of intracellular FH by influencing cell proliferation and migration was found at both the gene and protein levels in clear cell renal cell carcinoma and in lung adenocarcinoma. Intracellular FH conferred poor prognosis in patient cohorts with clear cell renal cell carcinoma and lung adenocarcinoma, but not in patients with lung squamous cell carcinoma (154). Autoantibodies against FH were found in the sera from patients with early stage, non-metastatic NSCLC (155). These FH autoantibodies interfered with the C3b binding site of the molecule, thereby enhanced complement activation on tumor cells *in vitro* (156). The potential use of FH-based treatments in cancer therapy is an emerging and important practical topic. Monoclonal anti-FH antibody treatments have been used successfully *in vitro* and in a lung cancer mouse model *in vivo* (156, 157), and the FH monoclonal antibody is currently in a Phase Ib clinical trial for advanced lung cancer. In the case of B cell chronic lymphocytic leukemia cells *ex vivo* complement dependent cytotoxicity was enhanced by anti-FH when added together with rituximab (158). Beside monoclonal antibodies a recombinant FH derived protein composed of CCPs 18-20 (hSCR18-20) was successfully used to inhibit the binding of FH to chronic lymphocytic leukemia cells and an FHR-4—anti-HER2 immunoconjugate was able to induce complement dependent cytotoxicity on HER2 expressing tumor cells that are known to be resistant to complement mediated lysis (159–161).

Increased FH and FHL-1 expression of glioma patients was associated with poorer survival (162). It was also demonstrated that low levels of FHR-1 are specifically found in lung adenocarcinoma samples and downregulation of FHR-1 correlated with lower overall survival and post progression survival times (163). Gasque et al. described that neuroblastoma cell lines express FH as well, while primary tumor cells from glioblastoma multiforme patients were found to secrete FHR-5, but not FH (142, 164). This tumor cell derived FHR-5 inhibited complement mediated lysis, possessed co factor activity for factor I mediated cleavage and accelerated decay of C3 convertase (164). In other studies, FHR-5 expression was associated with the risk of metastasis in colorectal cancer and soft tissue sarcomas (165, 166). On the contrary, Koshiol et al. examined HBV- and HCV-related hepatocellular carcinoma (HCC) and found that FHR-5 was negatively associated with the development of HCC compared to non-cirrhotic controls (167). Also, FHR-4 expression was lower in HCC tumor tissue compared to normal tissue, and the lower expression of FHR-4 was associated with HCC progression and a poor prognosis (168). Liu et al. demonstrated that liver cancer tissue had lower FHR-3 mRNA and protein levels compared with normal tissue and overexpression of FHR-3 suppressed proliferation and promoted apoptosis (169). These findings are strengthened by the observation that elevated levels of FHR-3 was correlated with a good prognosis for HCC patients (170–172). Lack of complement regulation can also lead to tumor formation, since FH-/- mice were shown to develop spontaneous hepatic tumors and HCC patients with increased FH mRNA had better prognosis, while mutations of FH associated with worse survival (173). However, mutations in the *CFH* gene were associated with better response to immune checkpoint inhibitors, although only in men (174).

Tumor-derived extracellular vesicles (EVs) can also contain complement proteins and FH was found to be highly expressed in EVs released by metastatic HCC cell lines. These EVs promoted HCC cell growth, migration, invasiveness, and enhanced liver tumor formation in mice; these effects were abrogated when treated with an anti-FH antibody (175). EVs of metastatic NSCLC was found to be FH positive as well, while FHR-4 was downregulated in small-cell lung cancer samples’ EVs (176, 177).

Recently, a *CFH*-derived circular RNA (circRNA), a type of non-coding RNA, termed circ-CFH was reported and investigated in connection with glioma. Circ-CFH expression was significantly upregulated in glioma tissue and was correlated with tumor progression (178). **Table 1** summarizes the reports on FH and FHRs as potential biomarkers.

**Table 1:** Potential FH family biomarkers in different cancers.

Cancer	Potential biomarker	Reference
bladder	FH increased in urine and plasma	179, 180
oral cavity squamous cell carcinoma (OSCC)	higher salivary level of FH was correlated with advanced stages of OSCC	181
lung adenocarcinoma	FH-positive tumors had worse prognosis	152
lung adenocarcinoma and clear cell renal cell carcinoma	Intracellular FH conferred poor prognosis	154
small-cell lung cancer	upregulation of FHR-4 in EVs	177
hepatocellular carcinoma	FHR-3 overexpression was correlated with a good prognosis for HCC patients.	171
hepatocellular carcinoma thyroid cancer	Lower FHR-4 expression Increased FHR-1	168, 182
non-Hodgkin’s lymphoma	Increased FHR-3 (biomarker for obinutuzumab response)	183
cutaneous squamous-cell carcinoma	FH and FHL-1 were higher expressed in tumors	184

Expression of FH can lead to the downmodulation of complement activation in the tumor microenvironment, moreover, by binding to immune cells FH may also modulate the anti-tumor response (non-canonical role of FH). FH expression by breast cancer cells promotes tumor growth via immunosuppression and positively correlates with the presence of immunosuppressive macrophages, since FH directly promotes the differentiation of blood-derived monocytes into immunosuppressive macrophages (185). The complex role of the complement system in pro- and antitumor effects is reviewed by Roumenina et al. and the effect of (locally) elevated/downregulated FH and FHR proteins has to be integrated into this (146). FHRs as positive regulators of complement may help enhance complement attack on tumor cells but may also increase local inflammation which could promote tumorigenesis. In addition, in their noncanonical roles they may influence the activation of immune cells, potentially also that of tumor-associated neutrophils and macrophages. Therefore, further research is needed to reveal their exact role in oncological disorders, which in turn may be tumor- and context-dependent.

### Interaction of FH family proteins with microbes

Antimicrobial effects of complement include opsonization, the generation of anaphylatoxins and chemoattractants, and particularly in the case of gram-negative bacteria lysis through the formation of pores by the membrane attack complexes (186, 187). Microbes evolved to resist complement attack by different mechanisms including capsule production, the production of proteases cleaving the complement components, and recruitment of complement regulators to the microbial surface (188–191).

Several microbes bind FH and FHL-1 on their surface, such as *Borrelia burgdorferi, Staphylococcus aureus, Neisseria meningitidis*, *Haemophilus influenzae, Candida albicans* and others (51, 192–197). By recruiting the host regulators FH and FHL-1, they can downmodulate complement activation on their surface. Most pathogens recruit FH through CCP domains 6–7 and/or 18–20 that on host cells bind to glycosaminoglycans and to C3b and glycosaminoglycans, respectively, thereby permitting FH (and FHL-1) domains 1–4 to inhibit complement at the same time (198, 199). Identifying the molecular basis of FH binding by microbial proteins is important to understand how this immune evasion strategy of microbes works. Recently, the crystal structure of the FhbA protein of *Borrelia hermsii* in complex with FH domains 19-20 was determined. Based on the identified structural elements that are responsible for FH binding, new putative FH binding proteins were identified (200).

The protective role of FH was proved in some cases by knocking out or knocking in microbial genes that encode proteins responsible for FH binding. The *Aspergillus fumigatus* protein Aspf2 binds FH, FHL-1 and FHR-1. Conidia in which *aspf2* was knocked out had less FH and FHL-1 and, consequently, more deposited C3 fragments on their surface, compared to the wild-type conidia (201). Leptospiral Lig proteins that bind FH, FHL-1 and FHR-1 were introduced into the saprophytic *L. biflexa* which does not express these complement evading proteins by itself. This genetic modification allowed the bacteria to bind FH and C4BP and thereby prevented complement deposition on their surface (202). Similar observation was made by Marshall and colleagues when introducing the *pspc* gene of *Streptococcus pneumoniae* into the non-pathogenic, thereby serum-sensitive *Streptococcus mitis*, which then gained the ability to bind FH and reduce complement activation on its surface (203). There are, however, also contradictions within the literature regarding the relevance of FH binding. Studies on *N. meningitidis* showed that the recruitment of FH helped the bacteria to escape from complement attack (204–206), but a recent study showed that during acute meningococcal disease the serum levels of all FH family proteins are decreased and no specific FH depletion by *N. meningitidis* was observed (207). In the case of *Streptococcus pyogenes* FH binding did not show any benefit on the microbial survival *in vivo* (208). Moreover, FH when bound on *C. albicans* increased the cellular antifungal response (195).

Several microbes do not only bind FH, but different FHR proteins, as well. *B. burgdorferi* was shown to bind FHR-1, FHR-2, and FHR-5. These FHRs had no effect on complement evasion that suggested a cooperation with FH to escape complement attack (209–211); whether they in fact compete with FH and enhance complement activation was not investigated. Similarly, a construct generated from Staphylococcal Sbi protein domains III and IV enhanced the binding of FH, FHR-1, FHR-2, and FHR-5 to C3b fragments. At physiological concentrations FHR-1 was able to dissociate FH from C3b in the presence of Sbi-III-IV and this was associated with more pronounced complement activation (212). Competition between FH and FHR-1 for binding to *E. coli* shiga toxin 2 was shown, and FHR-1 inhibited FH binding in a dose-dependent manner (213). *Plasmodium falciparum* is a parasite that binds both FH and FHR-1, and competition between FHR-1 and FH for binding to the parasite occurs in this case, too (214).

*N. meningitidis* recruits FHR-3 that competes with FH for binding to the microbial surface and therefore, the survival of the microbe depends on the serum levels and ratio of FH and FHR-3 (215). Reduced level of FH was found to be a protective factor from meningococcal disease and the lower FH concentration was associated with a single nucleotide polymorphism in the *CFHR3* gene. Deletion of the corresponding gene segment in an engineered cell line elevated the FH level, thereby a direct regulatory role of this polymorphism was proved, although the exact mechanism is unclear (216). *N. meningitidis* and *N. gonorrhoeae* both bind FHR-5 by the same outer membrane protein PorB in the presence of sialylated lipopolysaccharide, and the addition of FHR-5 to sialylated *N. gonorrhoeae* caused enhanced complement-mediated lysis of the bacteria (217). The exact role of FHR-5 in host defense is yet elusive, however, low levels of FHR-5 were detected in two patients who had infection-associated aHUS and membranoproliferative glomerulonephritis (218).

Altogether, these results point to a protective role of several FHR proteins in infectious diseases by counteracting the host FH-sequestering complement evasion strategies; thus FHRs may developed as a response of the host to the FH mediated complement evasion of microbes (50, 51).

Viruses such as the Human Immunodeficiency Virus and West Nile Virus were shown to bind FH as well to evade complement (219, 220). FH polymorphisms and haplotypes that result in altered FH expression were found protective against severe dengue (221). Recently, the role of complement in the thromboinflammation caused by SARS-CoV-2 got significant attention. FH was found to be upregulated among many other complement- and coagulation related genes under high viral load (222); in addition, FH expression was upregulated in lung epithelial cells of COVID-19 patients (223). The spike protein of SARS-CoV-2 was shown to compete with FH for binding to heparan sulfate, causing complement dysregulation on the cell surface (224, 225). Serum proteomic analyses also detected increased FH and FHR-5 levels in patients infected with SARS-CoV-2 compared to healthy controls (226–228). FHR-2 elevation in serum was also described (229). In a recent study, serum levels of all FH family proteins in COVID-19 patients and controls were quantified using a targeted mass spectrometry approach; while no elevation in FH level was found, the serum levels of all other FH protein family members were increased, with FHR-2 and FHR-5 showing the highest elevation in severe COVID cases compared to controls (230). The authors raised the hypothesis that the elevated circulating FHR levels could predict disease severity of COVID-19 patients (230). These studies suggest that complement dysregulation associated with SARS-CoV-2 infection involves the disbalance among FH family proteins.

Binding of FH family proteins may not only influence complement activation but also direct interaction with immune cells. For example, the yeast *C. albicans* recruits FH, FHL-1, FHR-1, and FHR-4, where FH, FHL-1 and FHR-1 were shown to be major ligands for CR3 (CD11b/CD18) on neutrophils. Yeast-bound FH and FHR-1 were able to enhance the antimicrobial activity of neutrophils while the role of FHR-4 remained ambiguous (195). This direct action on immune cells relates to the non-canonical roles of members of the FH protein family that is discussed later.

## Development of FH-based therapeutics

Many microbes evade complement through the recruitment of complement regulators such as FH. Therefore, blocking FH binding to microbes could be a way to restore their sensitivity to human complement. It was shown in the case of *Streptococcus pyogenes* that addition of a recombinant FH construct containing CCP5-7 strongly reduced bacterial survival in human blood (231). In the case of *Neisseria* sp., a fusion protein was designed that is composed of FH domains CCP6-7 and/or CCP18-20 fused with the constant region of IgG1 (IgG1 Fc) to engage C1q, thereby inducing classical pathway activation by the formed FH6,7/Fc and FH18-20/Fc. The chimeric proteins caused complement dependent killing *in vitro* and showed high efficacy in *in vivo* studies on animal models of gonorrhea and meningococcal infections (232). The chimeric protein FH6,7/Fc against *N. meningitidis* was able to block FH binding to the bacteria, and enhanced C3 and C4 deposition that facilitated classical complement pathway mediated killing of meningococci; the same construct when applied in *in vivo* experiments on infant rats infected with serogroup C strain resulted in 100-fold reduction in infection compared to the control animals (233). In addition to *Neisseria* species, more work have been done on *H. influenzae* where it was shown that FH18-20/Fc was less effective in the promotion of complement mediated killing of the different strains than FH6,7/Fc; moreover, intranasal delivery of the chimeric protein in the lung infection model resulted in sufficient clearance of *H. influenzae* (234). The chimeric protein FH6,7/Fc was tested on Group A streptococcus as well. Sepsis was induced by the bacteria in a transgenic mouse model that expresses human FH, and FH6,7/Fc was able to enhance alternative pathway activation and reduce bacterial presence *ex vivo* through enhancement of opsonophagocytosis in a C3 dependent manner (235).

Currently, there are several therapeutic agents, including antibodies, recombinant proteins, peptides, and small molecules, in various clinical phases against complement-mediated diseases (236–238). FH based engineered inhibitors could have a great potential in the treatment of complement associated diseases to restore or enhance regulation of the cascade. Recombinant full-length FH was produced in various systems to replace the missing or functionally defective FH and/or enhance complement inhibition (239–242). In addition, constructs using parts of FH have been developed and characterized.

One of the most well-known FH derivates is MiniFH (243–246). MiniFH is >10-fold potent inhibitor of the complement alternative pathway than full length FH (244). This engineered inhibitor contains the two main functional segments of the native protein. At the N terminus there are the CCP1-4 (or CCP1-5) domains which are responsible for the complement regulatory functions of FH, the decay acceleration activity and the cofactor activity (247, 248). At the C terminus, domains CCP19-20 (or CCP18-20) provide C3b and GAG binding, which occur simultaneously (38, 39). When FH is in solution the C3b binding site could be masked by the CCPs between the two functional sites, which may be the reason why MiniFH (in which CCP5-CCP18 or CCP6-17 are deleted) is a stronger inhibitor than FH (244, 249). CCP19-20 contains the sialic acid binding site, allowing FH and MiniFH to discriminate host surfaces from other surfaces through the binding of GAGs (39). To enhance the MiniFH C3b binding capacity, a double MiniFH was generated, called MidiFH in which two MiniFHs are linked together with a linker region (249). The inhibitory potency of MidiFH in the alternative pathway is stronger than MiniFH, probably because the MidiFH contains doubled C3b binding sites (249). Both MiniFH and MidiFH can effectively inhibit alternative pathway on erythrocytes derived from patients with paroxysmal nocturnal hemoglobinuria (PNH), thereby these inhibitors can protect them from complement mediated lysis (246, 249). The effectiveness of inhibition by MiniFH could also be further increased by introducing dimerization domains of FHRs, e.g. FHR-1. These domains lead to formation of homodimers, which have a prolonged half-life; furthermore, this homodimeric MiniFH has an improved alternative pathway regulatory capacity. This chimeric MiniFH which contains the dimerization domains of FHR-1 had been tested *in vivo* in FH deficient mice in which the inhibitor restricted the accumulation of C3 in the glomeruli (250, 251). Taken together, different MiniFH constructs show promising results both *in vitro* and *in vivo*, thus giving hope for a future therapeutic agent. To date, MiniFH called AMY-201 (manufactured by Amyndas) with polyglicine linker between the two functional sites is in preclinical development (252, 253). Beyond this, MiniFH could modulate the complement-dependent release of IL-6 and IL-10 in a peripheral blood mononuclear cell model, and thus it may modulate the cytokine storm (254).

A different approach for complement regulators engineered from FH derivates is to create fusion proteins to optimize their complement regulatory potency or to increase their half-life. The construct CRIg/FH contains the CRIg extracellular domain, which can bind to C3 degradation products (C3b, iC3b, C3c) linked with the alternative pathway regulatory CCP1-5 domains of FH. CRIg/FH binds to sites in the body where complement activation is ongoing and shows complement inhibitory effects. This was demonstrated on PNH erythrocytes and in complement-mediated membranoproliferative glomerulonephritis in experimental rat models (255). In a recent study, CRIg/FH ameliorated lupus nephritis in a mouse model (256).

A single chain (scFv) B4 antibody -which is a mouse autoreactive IgM antibody- was linked to the alternative pathway regulatory segment of FH in the construct B4-scFv-fH. Antibody B4 targets an annexin IV neoepitope which is exposed upon oxidative stress and/or smoke exposure in animal AMD models; B4-scFv-fH effectively reduced complement activation in two mouse models of retinal degeneration, suggesting that it may be a promising candidate for a future therapeutic agent (257). Similarly, a mouse study was performed with IgG-FH_1-5_, where a non-targeting mouse IgG was coupled to the FH alternative pathway regulatory region (CCP1-5). In FH deficient mice, the IgG-FH_1-5_ was able to reduce C3 deposition in the kidney glomeruli, thus this inhibitor may serve as potential therapeutics in C3G. Moreover, due to the IgG part, the half-life of the molecule increased up to 11 days (258).

TT30 is a complement receptor 2 (CR2) and FH fusion protein, which contains the CCP1-2 domains of CR2 that bind to the C3 breakdown fragments iC3b, C3dg, and C3d generated at sites of complement activation. The functional site is fused with the FH regulatory segment CCP1-5 via CR2 CCP3-4 (259). TT30 appeared a promising therapeutic agent in several alternative pathway mediated complement disease models, such as AMD and PNH (260–262). However, complement inhibition by MiniFH was more effective than TT30 on PNH erythrocytes (246). In addition, TT30 is an effective inhibitor of both C3 and C5 convertases. Furthermore, in cynomolgus monkeys, TT30 was able to achieve an almost full alternative pathway inhibition for up to 24h (263). However, while TT30 was in clinical phase I for PNH, it did not progress beyond that (253).

A further engineered FH derivative is a dual inhibitor of the alternative and lectin pathways, called sMAP-FH. sMAP (small MBL-associated protein) is an alternatively spliced variant of MASP-2, composed of CUB and EGF-like domains, but lacking the serine protease domain of MASP-2. It can form complexes with the pattern recognition molecules of the lectin pathway (MBL, ficolins), thus it has a regulatory role in the lectin pathway activation via competing with MASP-1 and MASP-2 for ligand binding. In sMAP-FH the sMAP domains are linked to FH CCP1-5 and the construct showed strong inhibitory effect on both alternative and lectin pathway activation *in vitro* in mouse and human serum; moreover, it also reduced complement activation in mice *in vivo* (264).

Altogether, these FH-derived and hybrid recombinant proteins have shown promising results both *in vitro* and *in vivo* in regulating complement activation. Further preclinical studies and clinical trials will show if any of these can become a marketed drug.

## Animal models to study FH and FHR functions and their therapeutic applications

Although the scientific community has already raised concerns about the use of animals in research because of their welfare and the accuracy of outcomes, animal models of different human diseases are still used to obtain invaluable information about disease prevention, diagnosis, and possible therapeutic interventions. Using mice as model animals to investigate the involvement of complement components in human diseases generated important insights, even though there are differences between the human and the mouse complement systems (265). Although mouse FH (mFH) is very similar in structure and function to human FH, clear differences exist as well (49, 265). For example, in contrast to the human *CFH* gene, the *mCfh* gene does not have an alternative splicing variant and thus no murine equivalent of FHL-1 has been identified (49). Contrary to the situation in humans, murine FH was proposed to serve as immune adherence receptor on platelets (266). Furthermore, the *CFHR* genes have emerged during evolution through partial duplications of the *CFH* gene, and these events occurred after the separation of rodent and primate lineages and, therefore, no human FHR orthologs exist in mice (49, 59, 267). Thus, these members of the FH protein family have barely been examined in animal models. Inspiringly, new opportunities arise by which the elucidation of disease mechanisms and improved drug development become possible for instance by the establishment of genetically engineered humanized mice (49, 268).

To study the role of FH in the process of membranoproliferative glomerulonephritis which occurs in FH-deficient humans and pigs, Pickering and his colleagues generated the FH-deficient mice (fh-/- mice) by targeted disruption of the gene encoding murine factor H (269–272). Mice homozygous for the disrupted allele were viable and fertile and were analyzed on a hybrid, 129/Sv × C57BL/6 genetic background. The results clearly showed that without the expression of FH, Cfh-/- mice developed membranoproliferative glomerulonephritis spontaneously and were hypersensitive to developing renal injury caused by immune complexes; moreover, these animals developed spontaneous hepatic tumors as mentioned earlier (173, 272). The same knockout mice were backcrossed onto C57BL/6 mice and used in a recent study to analyze the role of FH in the physical performance of the animals. The study demonstrated for the first time the important role of FH and the alternative pathway of the complement system in physical performance and skeletal muscle health (273).

The contribution of FH to AMD pathogenesis was analyzed using different existing complement models including the already mentioned genetically modified mice (272). In their study, Coffey et al. analyzed aged, FH-deficient mice, which exhibited significantly reduced visual acuity and rod response amplitudes on electroretinography compared with age-matched controls (274). They also examined tissue sections from the neural retina and found an accumulation of complement C3, thinning of Bruch’s membrane, and disorganization of rod photoreceptor outer segments. Together with a subsequent study, which similarly showed that C3 and C3b are progressively deposited on retinal vessels leading to a reduction of retinal blood supply, these experiments demonstrated the importance of FH in the long-term functional health of the retina (274, 275). Although the reported ocular phenotype in this mouse model does not replicate a key AMD feature, sub-RPE (retinal pigment epithelium) deposits, these aging FH knockout animals developed retinal abnormalities and visual dysfunction, resembling AMD (276).

Animal models also proved to be helpful to decipher the functions of specific domains within FH. Loss of the CCP16-20 region of the protein impairs the ability of FH to control complement activation on surfaces, causing spontaneous complement-mediated thrombotic microangiopathy (49, 277).

Murine equivalents of FHR proteins are predicted based on sequence analysis and are termed FHR-A to FHR-E (278). The *in vivo* expression and function of these proteins is largely unknown as well as their relevance to their human counterparts. They show some similarities to the human FHRs, although murine FHRs show higher sequence identity to mFH than do the human proteins to human FH, which suggests that results with mouse FHRs cannot be directly translated to the human system. Despite these challenges, some data are available regarding mouse FHR proteins. Recombinant murine FHR-B was shown to act similarly to human FHR-1 and FHR-5, namely, to enhance complement activation on PTX3, CRP, the ECM model Matrigel and necrotic cells (279). Parallel to the human system, FHR-A and FHR-B were shown to increase complement activation by competing with mouse FH for ligand binding, while FHR-C did not have such effect. Exact K_D_ values for C3d binding were determined for each member of the murine FH family that correlated well with the deregulating effect of FHR-A and FHR-B (280). FHR-E deficient mice showed elevated complement activation of the alternative pathway induced by LPS and had acute kidney injury, and the authors suggested that this mouse may be a model to study human FHR-1 deficiency related alternative pathway overactivation (281). A recent study investigated the role of recombinant FHR-A, FHR-B, FHR-C and FHR-E and demonstrated differential interactions of these murine FHRs with FH ligands such as heparin and C3d; however, all four FHRs caused complement dysregulation *in vitro* on the surface of renal cells. *In vivo*, only FHR-E caused complement dysregulation on glomerular cells in a mouse ischemic acute kidney injury model (282).

A specific model for CFHR5 nephropathy was also developed, in which mice lacking mFH and murine FHRs but coexpressing human FH and mutant FHR-5 (with duplicated CCP1-2) developed glomerular deposition of C3 fragments, while those coexpressing human FH and wild type FHR-5 did not (283). In addition, administering mutant FHR-5 to the latter mice also caused C3 deposition in the glomeruli. Thus, the results recapitulate the human situation and may allow for the evaluation of potential therapeutic interventions for this specific disease (283).

These results give hope that with better characterization of murine FH family members and the generation of transgenic mice, the use of mouse disease models will be available to study the pathologic role of both FH and FHRs in detail. Moreover, FH-based and other therapeutic agents could be tested *in vivo* in such models. These models may also result in the discovery of novel roles and mechanisms of these proteins, including non-canonical roles.

## Non-canonical roles of FH family proteins

Beyond its role in modulating complement activation, FH has been reported to bind to various immune cells and regulate their functions known as the non-canonical role of the molecule. This binding is mediated by the complement receptors CR3 (CD11b/CD18) and CR4 (CD11c/CD18) (195, 197, 284–287), although other potential receptors for FH cannot be ruled out, as in a study FH was shown to directly bind to Siglec-9 (288). In addition, we must consider that the interaction of FH with the cell surface is also influenced by GAGs, sialic acid and other ligands deposited due to complement activation, such as complement C3 fragments, thus CR3/CR4 cis-ligation may also occur on cell surfaces. Nevertheless, cells can bind FH in the absence of deposited C3b (289–291), but the receptor bound C3 fragment may potentially modify the interaction of FH with cells. In the absence of C3b, FH binding to CR3 expressing monocytes can be inhibited by apolipoprotein E (apoE), which indicates a common binding site for apoE and CR3 on FH, although complement receptor CR1 (CD35) and CD89 were also suggested to be involved in FH binding (290, 292). Interestingly the receptor-bound FH retains its cofactor activity for C3b inactivation (287). The exact binding site of FH on CR3 is unknown, however, published data suggest that FH and iC3b bind at different sites to the receptor (287, 293, 294); thus, the effects they induce may be combined rather than competing. In contrast to FH, cell surface receptors for FHR proteins are more elusive. FHR-1 was identified as a ligand of CR3 (195), also that of EMR2, and at high concentration, FHR-1 but not FHR-5 was shown to significantly decrease binding of FH to immune cells, indicating that FH and FHR-1 have partially overlapping binding sites (291, 295).

FH family members act as circulating molecular sentinels that continuously monitor for altered structures or invading pathogens to which they can attach and then modulate complement activation and cellular functions. They exert their cellular modulatory role mainly when they are deposited to a surface (287, 291, 296), thus the *in vivo* relevance of the non-canonical FH/FHR functions is presumably local immune regulation. FH is produced not only in the liver, but its extrahepatic sources are known as well, which may underline its local importance. Myeloid dendritic cells in tissues and endothelial cells can produce FH upon inflammatory stimuli; monocytes and B cell lines were also described to produce FH or FH-related protein (297–304). Local production of FHR-3 by retina microglia/macrophages was observed, while primary tumor cells can be a source of FHR-5 (164, 305). The concept that local „niches” where the production and (canonical or non-canonical) function of complement proteins are unique and important is nicely exemplified by the finding that FHR-3 that is produced locally in the eye is internalized by the RPE cells (while FH is not). FHR-3 deregulated FH from oxidative stress-associated epitopes and induced the secretion of proinflammatory mediators in RPE cells through the activation of the NLRP3 inflammasome (306). Production of C3 and FB was also upregulated upon FHR-3 treatment and their cleavage products appeared inside and on the surface of the RPE cells; moreover, the expression of the complement receptors C3aR and CR3 was enhanced (306). Interestingly, in hepatocellular carcinoma the endogenous FHR-3 expression promoted apoptosis in the tumor cells and was associated with a good prognosis (169, 171). These findings underline that the effect of FH family members can largely depend on the local microenvironment and may be cell specific. Intracellular expression of complement proteins is a novel but emerging field giving new perspective to the understanding of the non-canonical roles of complement.

The role of intracellular FH in mouse (C57BL/6J) renal endothelial cells was studied using FH deficient and sufficient cells. In the absence of FH, NF-kB translocated to the nucleus showing that endogenous FH expression has an anti-inflammatory role, although increased angiogenesis also occurred in FH deficient kidney endothelial cell cultures. The FH-/- kidney endothelial cells had altered actin cytoskeleton organization and lost their barrier function as well. This latter important observation was strengthened in human endothelial cells as well where siRNA of FH reduced the barrier function (307). The role of intracellular FH expression was extensively studied in RPE cells with similar results. Silencing FH in RPE cells caused elevated C3, FB and FI production, inflammation, mitochondrial impairment and enhanced the sensitivity of the cells to oxidative stress. The activation of NF-kB p65 and the mTOR pathway was found to mediate these effects (308–310). In addition, silencing of FH in human RPE cells induced direct metabolic change and degeneration of retinal rod cells when cocultured with porcine retinal explant (311). These findings reveal unexpected roles and emphasize the complex operation of the complement system.

Daugan et al. (154) found that FH is present in human renal cell carcinoma tumor cells, moreover, its elevated level correlates with bigger tumor size and worse disease prognosis. The serum level of FH was the same in tumor patients and in controls, which clearly implicates that local, intracellular expression of FH has a profound role in shaping the phenotype of tumor cells. This was proved using clear cell renal cell carcinoma cell lines in which FH expression was reduced by RNA silencing. These FH silenced cells had downmodulated expression of genes linked to cell proliferation, which was confirmed in cell survival studies; moreover, extracellularly added FH could not reverse the effect (154). On the contrary, FH silencing had no effect in primary renal proximal tubule cells and in human umbilical vein endothelial cells that have FH expression normally, demonstrating that the observed effects are tumor specific and not universal. Interestingly, in the same study, B and T lymphocytes were found negative for FH, which suggests that FH plays a role mainly in the regulation of innate immune functions. Intracellular FH was localized in lysosomes (154), where C3 was also present, already shown to be a prosurvival signal (312). A putative mechanism of this protumor effect of elevated FH may be the initiation of anti-inflammatory conditions via binding to complement receptor CR3 (CD11b/CD18) on/inside innate immune cells, especially tumor associated macrophages. Similar anti-inflammatory action of FH was found in the case of apoptotic cells. The cells undergoing apoptosis internalized FH that contributed to their silent removal, probably by binding to nucleosomes and facilitating the generation of C3b fragments by cathepsin L and promoting the uptake of apoptotic cells via CR3 (288).

Although there is growing literature about the non-canonical role of FH, only a few data are available about the function of other FH family members. Since FHRs show different degree of sequence similarity and overlapping ligand specificity with FH, they are expected to have immunomodulatory effects as well. Monocytes and neutrophil granulocytes are the most extensively studied cell types regarding the non-canonical role of FH family proteins since both express the FH binding CR3 in high amounts. These circulating sentinel cells search for potentially dangerous structures in the blood and regulate inflammatory processes. FH affects almost every important cellular function of monocytes and neutrophils, and FHRs may fine-tune or modulate these effects depending on the available binding partners and the actual FH : FHR ratio. FH family proteins can serve as an adhesion ligand for neutrophils and monocytes and influence their spreading, an indispensable process of their extravasation and cell polarization (195, 287, 291, 313). Human neutrophils showed increased spreading over immobilized FH, FHR-1 and FHL-1, while in the case of monocytes only FH and FHR-1 elicited the same effect (291). FH can play a role in the recruitment of leukocytes to the site of damage/inflammation by having a chemotactic effect on monocytes and neutrophils on its own or when bound to *Candida albicans* or endothelial cells (195, 287, 293, 314–316). In addition, surface-bound but not soluble FH was shown to enhance the release of extracellular vesicles from neutrophils (317). The combination of FH with other stimuli may have different effect, since upon LPS mediated activation of monocyte-derived DCs (MoDCs), the FH treatment induced downregulation of CCR7 and reduced MoDC migration toward the chemokine CCL21, pointing to a tolerogenic, anti-inflammatory role of FH (318). Moreover, MoDCs exposed to FH had lower CD83 and CD86 expression and failed to stimulate allogeneic T cells, and produced decreased amounts of TNF-α, INF-γ, IL-8, and IL-6 and increased amount of IL-10, thus promoted adaptive Treg cell generation (318). These findings were confirmed in an other study where inhibition of FH production by DCs resulted in greater ability to induce the proliferation of allogeneic CD4+ T-cells (301). Similarly, FH-treated macrophages displayed immunosuppressive characteristics, including expression of CD163 and CD206, release of IL-10, low level expression of HLA-DR but high levels of the co-inhibitory molecule programmed death-ligand 1 (PD-L1) and, accordingly, showed a reduced capacity for T-cell activation (185). All these data point to a regulatory role for FH in the activation of antigen presenting cells by shifting their differentiation toward a tolerogenic phenotype; however, experimental results (pro- or anti-inflammatory effect) can depend on the context of FH treatment. Cells may respond differently depending on whether FH family proteins are in a soluble form or deposited on a surface.

**Figure 4:**
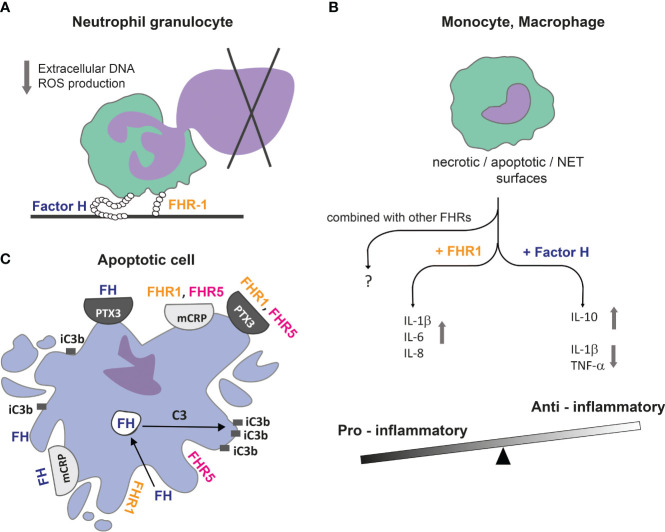
FH family proteins can balance between pro-inflammatory and anti-inflammatory conditions. **(A)** Immobilized FH and FHR-1 downregulate DNA / extracellular trap release from neutrophil granulocytes, contributing to reduced host damage caused by an inflammatory environment and the presence of autoantigens. **(B)** FH can bind directly to DNA or apoptotic and necrotic cells; together they induce an anti-inflammatory cytokine profile in monocytes and macrophages, while FHR-1 under the same conditions shifts processes into an inflammatory direction. **(C)** FH family proteins can influence complement activation and, therefore, the opsonisation pattern on dead cell surfaces. The actively internalized FH can promote C3 cleavage intracellularly in early apoptotic cells, resulting in cell surface accumulation of iC3b. Recruitment of FH by mCRP and PTX3 limits complement activation and facilitates removal of apoptotic cells in an anti-inflammatory manner. FHR-1 and FHR-5 can recruit both mCRP and PTX3 to necrotic cell surface and *vice versa*, and rather enhance complement activation (25).

FH was described to stimulate respiratory burst in monocytes and increase hydrogen peroxide generation by neutrophils upon C5a or TNF-α stimulation (284, 319). In contrast to this, FH was shown to inhibit anti-neutrophil cytoplasmic autoantibody (ANCA) mediated respiratory burst, as well as PMA or fibronectin plus β-glucan induced reactive oxygen species generation by neutrophils (287, 293). As mentioned earlier, silent removal of apoptotic cells is a vital process in which FH plays an important role (**Figures 4B, C**). Early apoptotic cells were found to bind and actively internalize FH to generate iC3b from endogenously expressed C3 by cathepsin L that supports safe and fast clearance of cell debris. In addition, internalized FH binds to nucleosomes and induces the production of IL-10 in monocytes (288). Mihlan et al. found that mCRP recruits FH to the apoptotic cell surface and in this combination, FH enhanced the uptake of these particles by macrophages and reduced TNF-α release, while Kang et al. did not observe the same effect (286, 320). FH has a significant contribution to the safe removal of apoptotic debris not only by preventing excess complement activation and generating iC3b, the main ligand of the phagocytic receptor CR3, but through its non-canonical, anti-inflammatory action on the main phagocytes, macrophages and neutrophils. Further investigations are needed whether this tolerogenic effect can be exploited by different therapeutic FH constructs in the treatment of chronic inflammatory diseases.

DNA can be exposed to the extracellular milieu not only during apoptosis, but through the formation of extracellular traps by neutrophils (NET) and monocytes (MoET) as well (321–324). It was shown that FH and FHR-1 could significantly decrease the amount of released extracellular DNA, as well as ROS production associated with NET release (287, 291). FH could bind to NET and MoET directly and the bound FH decreased the secretion of IL-1β in monocytes (293, 324). Based on these results, FH can reduce host damage caused by an inflammatory environment through the limitation of NET and proinflammatory cytokine production, which fits with the concept of its anti-inflammatory role (**Figure 4A**) (195, 287, 291, 296, 318).

In the case of monocytes, FH and FHR-1 rather enhanced IL-1β secretion, and FH also increased TNF-α release (291). However, the direct immunomodulatory effect of FH family proteins seems to be context dependent since they exert most significant effects when applied together with other stimuli, like TLR triggering. FH was shown to enhance proinflammatory cytokine production upon LPS treatment, while other studies found the opposite (291, 318). Both FHR-1 and FHR-3 were suggested to enhance inflammation, although FHR-1 and FHR-5 were shown to reduce proinflammatory cytokine production, too (291, 295, 296, 306, 325) (**Table 2**).

**Table 2:** FH family proteins alter the cytokine production.

Cell type	Factor H family protein	Activation stimulus	Cytokine	Effect	Reference
Neutrophil granulocyte	FH, FHR-1	–	IL-8	increase	287, 291
Monocyte	FH	–	TNF-α	increase	291
Monocyte	FH, FHR-1	–	IL-1β	increase	291
Monocyte	FH, FHL-1	LPS	TNF-α	increase	291
Monocyte	FHR-1, FHR-5	LPS	TNF-α	decrease	291
Monocyte	FHL-1	LPS	IL-10	increase	291
Monocyte	FHR-1	LPS	IL-1β, TNF-α, IL-6	increase	295
Monocyte	FHR-1	necrotic type surface	IL-1β, IL-6, IL-8	increase	296
Monocyte	FH	NET	IL-1β	decrease	324
Monocyte	FH	nucleosome, apoptotic cells	IL-10	increase	288
Monocyte	FH	mCRP in apoptotic cell surface	TNF-α	decrease	320
Monocytes from FHR-1/FHR-3 deficient donors	FH	LPS	TNF-α, IL-1β, IL-6, IL-10	increase	326
Macrophage	FH	*Candida albicans*	IL-1β, IL-6	increase	197
FH induced macrophage phenotype	FH	LPS	IL-10	increase	185
MoDCs	FH	LPS	IL-10	increase	318
MoDCs	FH	LPS	TNF-α, INF-γ, IL-6, IL-8,	decrease	318
retina pigment epithelial cells	FHR-3	–	IL-1β, IL-6, IL-18	increase	306

Regarding the adaptive immune system, only B cells are reported to bind FH, but the receptor has not been identified yet (327–329). FH was shown to induce the release of complement factor I from B cells and support their proliferation, but block differentiation and inhibit immunoglobulin secretion (330–332). FH deficiency results in enhanced BCR signalling, which results in abnormal splenic B cell development in mice and lead to B cell-dependent autoimmunity with increased levels of dsDNA autoantibodies (333). These data provide evidence for a role for FH in directly calibrating B cell responsiveness and limiting autoimmunity. In addition, the C3d-mediated coactivation of human B cells was inhibited by FHR-3 by blocking the interaction between C3d and CD21 (334).

Altogether, these older and recent data suggest that the non-canonical roles of FH and other FH family proteins are as relevant as their systemic complement regulatory and modulatory function. The published results summarized in this section, however, also highlight that much work must be done to clarify how FH family proteins are involved in the regulation of cellular functions and reconcile in part contradictory data, likely explained by their context-dependent roles.

## Summary and outlook

**Figure 5:**
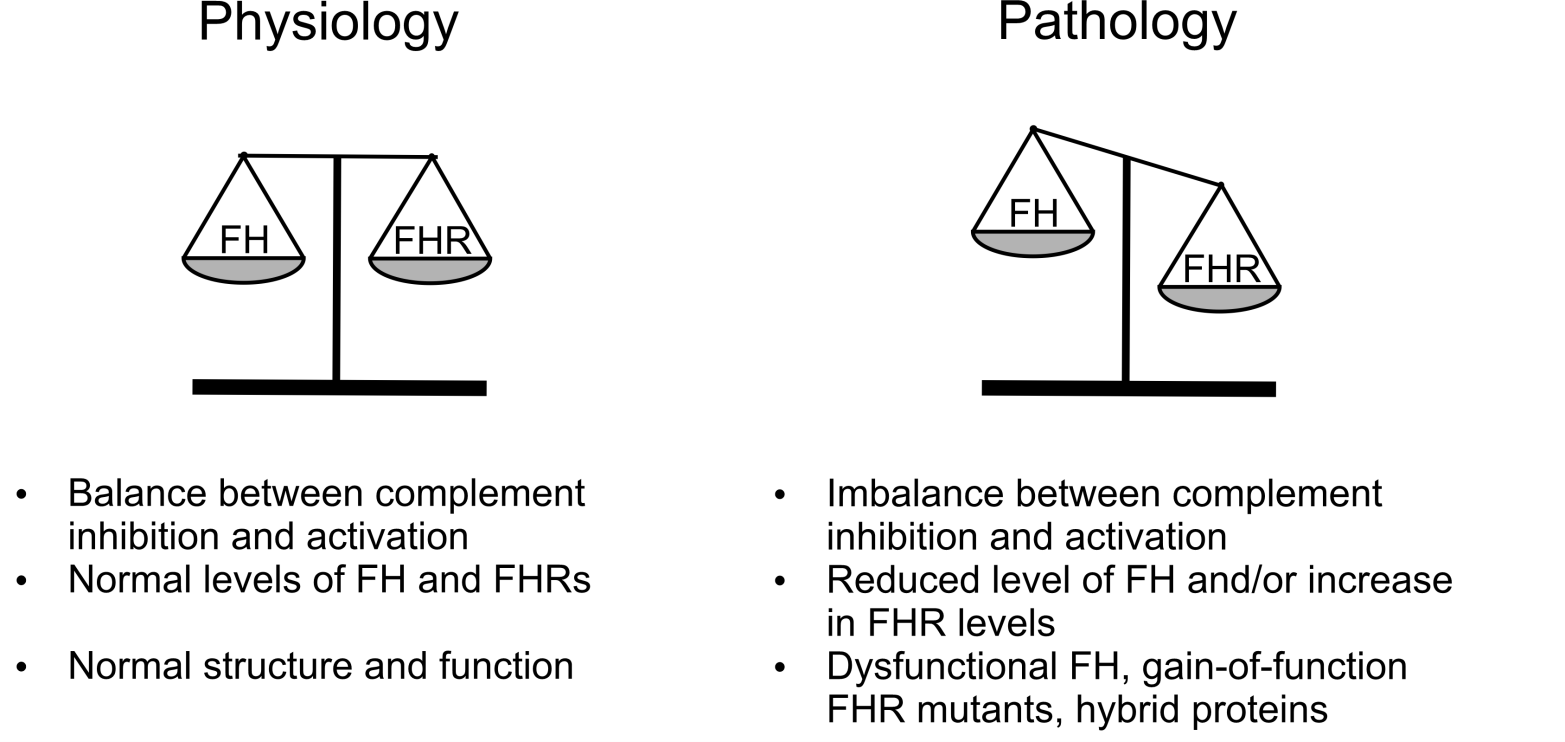
Role of FH family proteins in complement regulation. The state of balance between the inhibitor (FH) – and possibly that of FHL-1 – and the activator FHRs determines susceptibility to complement-mediated diseases. When inhibiton and activation are in an optimal balance (left panel), opsonization supported by the FHRs can proceed without the deleterious inflammatory and potential lytic activity of the terminal pathway. When levels or function of the proteins are altered in such a way that the balance is shifted towards overactivation (right panel), e.g. due to mutations or autoantibodies, the deleterious effects are enhanced and increase susceptibility to certain diseases.

As summarized here, the FH family proteins have a wide range of functions, in complement regulation and beyond. The roles identified thus far are consistent with a collaboration between FH and the FHRs in fine-tuning complement activation and facilitating optimal opsonization of target particles and surfaces (such as microbes, dying cells etc.). This model could accommodate the terminal pathway inhibiting effects of FHRs described in some reports. Shifting this balance towards too much activation due to altered FH : FHR ratios or function-affecting mutations and autoantibodies may result in pathological processes (**Figure 5**). The quantification of each member of this protein family in body fluids and the detection of them in tissues will be needed to understand their local roles more precisely. Measuring levels and performing genetic analyses will be particularly interesting in infectious diseases, neurological disorders, and cancer. Determining the concentration ranges and concentration changes for each FH family protein under physiological and inflammatory, pathological conditions will help the interpretation of the relevance of their competition and function *in vivo*, as well as the published *in vitro* results.

It would also be important to investigate which immune and non-immune cell types are able to produce FH family proteins *in vivo* and under which conditions, and to clarify the role of intracellular FH (and FHRs) in more detail under physiological and pathological conditions. Also, the cell surface receptors are to be studied, since CR3 alone cannot be responsible for all the non-canonical roles of FH, and the receptors of FHR proteins need to be identified.

Future research should focus in addition on structure-function studies, to identify relevant ligands and activities of these proteins; particularly the role of dimerization of some of the FHRs needs to be addressed. Understanding the formation and composition of FHR homo- and heterodimers and how they may change under various conditions has implications for their quantification as well as for their relevance in diseases.

With this information we can better understand physiological and pathological processes and hope to successfully translate this knowledge into therapeutic tools in order to restore the balance of FH and the FHR proteins and to ameliorate or prevent diseases.

## Author contributions

All authors listed have made a substantial, direct, and intellectual contribution to the work and approved it for publication.
