# Supplementary material for: The human factor H protein family – an update
Source: Front Immunol. 2024 Feb 12;15:1135490. doi: 10.3389/fimmu.2024.1135490 (PMC10894998; doi:10.3389/fimmu.2024.1135490)
Supplement: Supplementary file 1 [file DataSheet_1.pdf]

Supplementary Material

The human Factor H protein family – an update

Noémi Sándor[1,2], Andrea E. Schneider[1], Alexandra T. Matola[1], Veronika H. Barbai[1], Dániel Bencze[1], Hammad Hani Hashim[1], Alexandra Papp[1], Dorottya Kövesdi[1,2], Barbara Uzonyi[1,2], Mihály Józsi[1,2]

[1]Department of Immunology, ELTE Eötvös Loránd University, Budapest, Hungary
[2]HUN-REN-ELTE Complement Research Group, Hungarian Research Network, Budapest, Hungary

Corresponding author: Mihály Józsi, Department of Immunology, ELTE Eötvös Loránd University, Pázmány Péter sétány 1/c, H-1117 Budapest, Hungary; E-mail: mihaly.jozsi@ttk.elte.hu.

Supplementary Table 1. FH protein family alterations and their effects

Gene	Variation	Disease	Effect	Reference
CFH	Arg53Cys	AMD	Reduced cofactor activity; reduced decay accelerating activity; reduced inhibition of sC5b-9 on an AP-activating surface	Yu et al., 2014; Biggs et al., 2022
CFH	Arg53His	AMD	Reduced C3b binding, reduced decay accelerating activity; reduced cofactor; reduced inhibition of sC5b-9 on an AP-activating surface activity	Biggs et al., 2022; Pechtl et al., 2011
CFH	Arg53His, Ile62Val combined	AMD/DDD/aHUS	Reduced cofactor activity; reduced decay accelerating activity	Pechtl et al., 2011

CFH	Ser58Ala	AMD/HUS	Reduced FH plasma level; reduced decay accelerating activity; reduced cofactor activity	Merinero et al., 2018; de Jong et al., 2022
CFH	Ile62Val	AMD/DDD	Slightly reduced cofactor activity	Pechtl et al., 2011
CFH	Ile62Val, Arg78G	AMD/DDD/aHUS	Weak affnity for C3b; reduced cofactor activity; reduced decay accelerating activity	Pechtl et al., 2011; Noris et al., 2010
CFH	Cys66Tyr	IgAN	Low FH level/partial FH deficiency	Tortajada et al., 2017
CFH	Gly69Glu	MPGN/C3G/aHUS/AMD	Slightly decreased surface decay accelerating activity	Wong et al., 2020
CFH	Gln81Pro	MPGN/C3G/aHUS/AMD	Lack of C3b binding; decreased/no cofactor activity; reduced decay accelerating activity	Wong et al., 2020
CFH	Asp90Gly	AMD	Reduced cofactor activity	Yu et al., 2014
CFH	Asp130Asn	MPGN/C3G/aHUS/AMD	Slightly decreased surface decay accelerating activity; reduced cofactor activity	Wong et al., 2020, Biggs et al., 2022
CFH	Arg175Gln	AMD	Normal FH level; incresed C3bBbP level; reduced decay accelerating activity; reduced inhibition of sC5b-9 on an AP-activating surface reduced cofactor activity	de Jong et al., 2022; Biggs et al., 2022
CFH	Arg175Pro	AMD	Low FH level; weaker binding to C3b; no decay accelerating activity; reduced inhibition of sheep red blood cell hemolysis	Wagner et al., 2016; Biggs et al., 2022
CFH	Cys192Phe	AMD	Low FH level	Wagner et al., 2016
CFH	Ser193Leu	AMD	Reduced cofactor activity	Geerlings et al. 2016; de Jong et al., 2022
CFH	Ser193Leu, Arg303Gln combined	AMD	Elevated C3bBbP level	de Jong et al., 2022
CFH	Trp198Arg	C3G/aHUS	Reduced C3b binding capacity; impaired cofactor activity; reduced decay accelerating activity; reduced inhibition of sheep red blood cell hemolysis	Cserhalmi et al., 2017; Szarvas et al., 2016
CFH	Cys205Termination	IgAN	Partial FH deficiency	Tortajada et al., 2017
CFH	Ile221Val	AMD	Reduced cofactor activity	Biggs et al., 2022
CFH	Arg303Trp	AMD	Reduced inhibition of sheep red blood cell hemolysis	Biggs et al., 2022
CFH	Gln400Lys	AMD/HUS	Normal/reduced FH level	Dragon-Durey et al., 2004; de Jong et al., 2022

CFH	Tyr402His	AMD/DDD	Decreased binding to CRP, heparin; reduced cellular debris clearence	Edwards et al., 2005; Haines et al., 2005; Hagemann et al., 2005; Klein et al., 2005; Zareparsi et al., 2005; Herbert et al., 2007; Laine et al., 2007; Montes et al., 2008
CFH	Pro503Ala	AMD	Reduced inhibition of sC5b-9 on an AP-activating surface; reduced inhibition of sheep red blood cell hemolysis	Biggs et al., 2022
CFH	Asn516Lys	aHUS	No data	Le Quintrec et al., 2008; Noris et al., 2010
CFH	Arg576Gly	AMD	Decreased binding to C3b; reduced decay accelerating activity; reduced inhibition of sC5b-9 on an AP-activating surface; reduced inhibition of sheep red blood cell hemolysis	Biggs et al., 2022
CFH	Ser714Termination	AMD, HUS	Normal/reduced FH level	Neumann et al., 2003; de Jong et al., 2022
CFH	Glu936Asp	aHUS	No data	Noris et al., 2010
CFH	Gln950His	aHUS	Reduced inhibition of sheep red blood cell hemolysis	Mohlin et al., 2015; Szarvas et al., 2016
CFH	Trp978Leu	IgAN	Decreased FH and C3 levels	Tortajada et al., 2017
CFH	Gln1137Leu	aHUS	No data	Noris et al., 2010
CFH	Glu1172Termination	HUS	Truncated protein; weaker C3b binding; no C3d binding; weaker binding to endothelial cells; reduced surface bound cofactor activity; reduced inhibition sheep red blood cell hemolysis	Heinen et al., 2007
CFH	Trp1183Leu	AHUS/DDD/AMD	Reduced C3b and C3d binding; reduced functional activity	Noris et al., 2010; Loeven et al., 2016
CFH	Thr1184Arg	AHUS/DDD/AMD	Increased heparin binding; increased binding to mouse glomerular epithelial cells	Loeven et al., 2016
CFH	Ser1191Leu	aHUS	Reduced inhibition of human and sheep red blood cell hemolysis	Noris et al., 2010; Herbert et al., 2012; Mohlin et al., 2015
CFH	Ser1191Leu, Val1197Ala combined	aHUS	Reduced inhibition of human and sheep red blood cell hemolysis	Noris et al., 2010; Herbert et al., 2012
CFH	Gly1194Asp	AMD/aHUS	Reduced decay accelerating activity; reduced inhibition of sC5b-9 on an AP-activating surface activity; strong inhibition of sheep red blood cell hemolysis	Biggs et al., 2022; Noris et al., 2010

CFH	Val1197Ala	aHUS	Reduced inhibition of human and sheep red blood cell hemolysis	Noris et al., 2010; Herbert et al., 2012; Mohlin et al., 2015
CFH	Glu1198Ala	AHUS/DDD/AMD	Increased heparin binding; increased binding to mouse glomerular epithelial cells	Noris et al., 2010; Loeven et al., 2016
CFH	Val1200Leu	aHUS	No data	Noris et al., 2010
CFH	Arg1210Ala	AHUS/DDD/AMD	Reduced heparin binding; reduced binding to mouse glomerular epithelial cells	Loeven et al., 2016
CFH	Arg1210Cys	aHUS/C3G/AMD	Low affinity C3b binding; weak binding to endothelial cells; covalent interaction with human serum albumin; reduced decay accelerating activity	Józsi et al., 2006; Sanchez-Corral et al., 2002; Recalde et al., 2016; Servais et al., 2012; Biggs et al., 2022
CFH	Arg1215Gln	AHUS/DDD/AMD	Reduced heparin binding; reduced functional activity	Noris et al., 2010; Loeven et al., 2016
CFH	Hybrid gene	aHUS	Association with aHUS	Venables et al., 2006
CFH/CFHR1	$FHR1_{1-3}$-FH_{19-20} hybrid protein	aHUS	Predisposition of aHUS	Eyler et al., 2013
CFH/CFHR3	FH_{1-19}-FHR3 hybrid protein	aHUS	Enhanced heparin binding; reduced decay accelerating activity	Francis et al., 2012
CFH/CFHR3	FH_{1-17}-FHR3 hybrid protein	aHUS	Impaired surface binding; reduced decay accelerating activity; reduced cofactor activity	Challis et al., 2016
CFHR1	Leu290Ser, Ala296Val combined	aHUS	Decreased affinity for C3b binding; stronger syalic acid binding; stronger competition with FH for ligand binding	Dopler et al., 2021; Martin Merinero et al., 2021
CFHR1	Leu290Val	aHUS	Increased binding to surface bound C3	Martin Merinero et al., 2021
CFHR1	$FHR1_{1-4}$-FHR1	C3GN	Increased interaction with surface bound C3b, iC3b, and C3dg; enhanced competition with FH	Tortajada et al., 2013
CFHR1	*CFHR1*B* gene variant; Tyr_{157}-Val_{159}-Gln_{175}	aHUS	Increased binding to C3b; increased binding to necrotic cells; increased competition with FH for C3b; enhanced IL-1beta and IL-6 secretion by monocytes; deregulatory effect on FH mediated C3 convertase regulation	Abarrategui-Garrido et al., 2009; Xu et al., 2022
CFHR2, CFHR5	$FHR2_{1,2}$-FHR5 hybrid protein	DDD	Low FHR2, FHR5 protein plasma level; competition with FH for C3b binding; enhanced C3 convertase formation and stability	Chen et al., 2014
CFHR2, CFHR5	$FHR5_{1-2}$-FHR2 hybrid protein	C3GN	Increased guinea pig red blood cell hemolysis	Xiao et al., 2016
CFHR3	*CFHR3*B* gene variant; c.721C>T	aHUS	No data	Barnabeu-Herrero et al., 2015

CFHR3, CFHR1	Deletion	AMD/IgAN	Protective; no competition with FH	Hughes et al., 2006; Alic et a., 2020; Gharavi et al., 2011; Tortajada et al., 2017; Medjeral-Thomas et al., 2017
CFHR3/CFHR1	FHR3$_{1-2}$-FHR1 hybrid protein	C3G	No data	Malik et al., 2012
CFHR5	Val170Met	IgAN	Enhanced C3b binding	Zhai et al., 2016
CFHR5	Asn178Ser	IgAN	Enhanced C3b binding	Zhai et al., 2016
CFHR5	Leu259Termination	IgAN	Decreased C3b binding	Zhai et al., 2016
CFHR5	Glu274Asp	IgAN	Enhanced C3b binding	Zhai et al., 2016
CFHR5	FHR5$_{1-2}$-FHR5 hybrid protein	C3G	No data	Medjeral-Thomas et al., 2014

Abbreviations: AMD: age related macular degeneration; aHUS: atypical hemolytic uremic syndrome; IgAN: IgA nefropathy; C3G: C3 glomerulopathy; C3GN: C3 glomerulonephritis; DDD: dense deposit disease

References:

Abarrategui-Garrido, C., Martínez-Barricarte, R., López-Trascasa, M., de Córdoba, S. R., & Sánchez-Corral, P. (2009). Characterization of complement factor H-related (CFHR) proteins in plasma reveals novel genetic variations of CFHR1 associated with atypical hemolytic uremic syndrome. Blood, 114(19), 4261–4271. https://doi.org/10.1182/blood-2009-05-223834

Alic, L., Papac-Milicevic, N., Czamara, D., Rudnick, R. B., Ozsvar-Kozma, M., Hartmann, A., Gurbisz, M., Hoermann, G., Haslinger-Hutter, S., Zipfel, P. F., Skerka, C., Binder, E. B., & Binder, C. J. (2020). A genome-wide association study identifies key modulators of complement factor H binding to malondialdehyde-epitopes. Proceedings of the National Academy of Sciences of the United States of America, 117(18), 9942–9951. https://doi.org/10.1073/pnas.1913970117

Bernabéu-Herrero, M. E., Jiménez-Alcázar, M., Anter, J., Pinto, S., Sánchez Chinchilla, D., Garrido, S., López-Trascasa, M., Rodríguez de Córdoba, S., & Sánchez-Corral, P. (2015). Complement factor H, FHR-3 and FHR-1 variants associate in an extended haplotype conferring increased risk of atypical hemolytic uremic syndrome. Molecular immunology, 67(2 Pt B), 276–286. https://doi.org/10.1016/j.molimm.2015.06.021

Biggs, R. M., Makou, E., Lauder, S., Herbert, A. P., Barlow, P. N., & Katti, S. K. (2022). An Evaluation of the Complement-Regulating Activities of Human Complement Factor H (FH) Variants Associated With Age-Related Macular Degeneration. Investigative ophthalmology & visual science, 63(12), 30. https://doi.org/10.1167/iovs.63.12.30

Challis, R. C., Araujo, G. S., Wong, E. K., Anderson, H. E., Awan, A., Dorman, A. M., Waldron, M., Wilson, V., Brocklebank, V., Strain, L., Morgan, B. P., Harris, C. L., Marchbank, K. J., Goodship, T. H., & Kavanagh, D. (2016). A De Novo Deletion in the Regulators of Complement Activation Cluster Producing a Hybrid Complement Factor H/Complement Factor H-Related 3 Gene in Atypical Hemolytic Uremic Syndrome. Journal of the American Society of Nephrology : JASN, 27(6), 1617–1624. https://doi.org/10.1681/ASN.2015010100

Chen, Q., Wiesener, M., Eberhardt, H. U., Hartmann, A., Uzonyi, B., Kirschfink, M., Amann, K., Buettner, M., Goodship, T., Hugo, C., Skerka, C., & Zipfel, P. F. (2014). Complement factor H-related hybrid protein deregulates complement in dense deposit disease. Journal of Clinical Investigation, 124(1), 145–155. https://doi.org/10.1172/JCI71866

Cserhalmi, M., Uzonyi, B., Merle, N. S., Csuka, D., Meusburger, E., Lhotta, K., Prohászka, Z., & Józsi, M. (2017). Functional Characterization of the Disease-Associated N-Terminal Complement Factor H Mutation W198R. Frontiers in immunology, 8, 1800. https://doi.org/10.3389/fimmu.2017.01800

de Jong, S., Tang, J., & Clark, S. J. (2023). Age-related macular degeneration: A disease of extracellular complement amplification. Immunological reviews, 313(1), 279–297. https://doi.org/10.1111/imr.13145

Dopler, A., Stibitzky, S., Hevey, R., Mannes, M., Guariento, M., Höchsmann, B., Schrezenmeier, H., Ricklin, D., & Schmidt, C. Q. (2021). Deregulation of Factor H by Factor H-Related Protein 1 Depends on Sialylation of Host Surfaces. Frontiers in immunology, 12, 615748. https://doi.org/10.3389/fimmu.2021.615748

Dragon-Durey, M. A., Frémeaux-Bacchi, V., Loirat, C., Blouin, J., Niaudet, P., Deschenes, G., Coppo, P., Herman Fridman, W., & Weiss, L. (2004). Heterozygous and homozygous factor h deficiencies associated with hemolytic uremic syndrome or membranoproliferative glomerulonephritis: report and genetic analysis of 16 cases. Journal of the American Society of Nephrology : JASN, 15(3), 787–795. https://doi.org/10.1097/01.asn.0000115702.28859.a7

Edwards, A. O., Ritter, R., 3rd, Abel, K. J., Manning, A., Panhuysen, C., & Farrer, L. A. (2005). Complement factor H polymorphism and age-related macular degeneration. Science (New York, N.Y.), 308(5720), 421–424. https://doi.org/10.1126/science.1110189

Eyler, S. J., Meyer, N. C., Zhang, Y., Xiao, X., Nester, C. M., & Smith, R. J. (2013). A novel hybrid CFHR1/CFH gene causes atypical hemolytic uremic syndrome. Pediatric nephrology (Berlin, Germany), 28(11), 2221–2225. https://doi.org/10.1007/s00467-013-2560-2

Francis, N. J., McNicholas, B., Awan, A., Waldron, M., Reddan, D., Sadlier, D., Kavanagh, D., Strain, L., Marchbank, K. J., Harris, C. L., & Goodship, T. H. (2012). A novel hybrid CFH/CFHR3 gene generated by a microhomology-mediated deletion in familial atypical hemolytic uremic syndrome. Blood, 119(2), 591–601. https://doi.org/10.1182/blood-2011-03-339903

Geerlings, M. J., de Jong, E. K., & den Hollander, A. I. (2017). The complement system in age-related macular degeneration: A review of rare genetic variants and implications for personalized treatment. Molecular immunology, 84, 65–76. https://doi.org/10.1016/j.molimm.2016.11.016

Gharavi, A. G., Kiryluk, K., Choi, M., Li, Y., Hou, P., Xie, J., Sanna-Cherchi, S., Men, C. J., Julian, B. A., Wyatt, R. J., Novak, J., He, J. C., Wang, H., Lv, J., Zhu, L., Wang, W., Wang, Z., Yasuno, K., Gunel, M., Mane, S., … Lifton, R. P. (2011). Genome-wide association study identifies susceptibility loci for IgA nephropathy. Nature genetics, 43(4), 321–327. https://doi.org/10.1038/ng.787

Hageman, G. S., Anderson, D. H., Johnson, L. V., Hancox, L. S., Taiber, A. J., Hardisty, L. I., Hageman, J. L., Stockman, H. A., Borchardt, J. D., Gehrs, K. M., Smith, R. J., Silvestri, G., Russell, S. R., Klaver, C. C., Barbazetto, I., Chang, S., Yannuzzi, L. A., Barile, G. R., Merriam, J. C., Smith, R. T., … Allikmets, R. (2005). A common haplotype in the complement regulatory gene factor H (HF1/CFH) predisposes individuals to age-related macular degeneration. Proceedings of the National Academy of Sciences of the United States of America, 102(20), 7227–7232. https://doi.org/10.1073/pnas.0501536102

Haines, J. L., Hauser, M. A., Schmidt, S., Scott, W. K., Olson, L. M., Gallins, P., Spencer, K. L., Kwan, S. Y., Noureddine, M., Gilbert, J. R., Schnetz-Boutaud, N., Agarwal, A., Postel, E. A., & Pericak-Vance, M. A. (2005). Complement factor H variant increases the risk of age-related macular degeneration. Science (New York, N.Y.), 308(5720), 419–421. https://doi.org/10.1126/science.1110359

Herbert, A. P., Deakin, J. A., Schmidt, C. Q., Blaum, B. S., Egan, C., Ferreira, V. P., Pangburn, M. K., Lyon, M., Uhrín, D., & Barlow, P. N. (2007). Structure shows that a glycosaminoglycan and protein recognition site in factor H is perturbed by age-related macular degeneration-linked single nucleotide polymorphism. The Journal of Biological Chemistry, 282(26), 18960–18968. https://doi.org/10.1074/jbc.M609636200

Herbert, A. P., Kavanagh, D., Johansson, C., Morgan, H. P., Blaum, B. S., Hannan, J. P., Barlow, P. N., & Uhrín, D. (2012). Structural and functional characterization of the product of disease-related factor H gene conversion. Biochemistry, 51(9), 1874–1884. https://doi.org/10.1021/bi201689j

Heinen, S., Józsi, M., Hartmann, A., Noris, M., Remuzzi, G., Skerka, C., & Zipfel, P. F. (2007). Hemolytic uremic syndrome: a factor H mutation (E1172Stop) causes defective complement control at the surface of endothelial cells. Journal of the American Society of Nephrology : JASN, 18(2), 506–514. https://doi.org/10.1681/ASN.2006091069

Hughes, A. E., Orr, N., Esfandiary, H., Diaz-Torres, M., Goodship, T., & Chakravarthy, U. (2006). A common CFH haplotype, with deletion of CFHR1 and CFHR3, is associated with lower risk of age-related macular degeneration. Nature Genetics, 38(10), 1173–1177. https://doi.org/10.1038/ng1890

Józsi, M., Heinen, S., Hartmann, A., Ostrowicz, C. W., Hälbich, S., Richter, H., Kunert, A., Licht, C., Saunders, R. E., Perkins, S. J., Zipfel, P. F., & Skerka, C. (2006). Factor H and atypical hemolytic uremic syndrome: mutations in the C-terminus cause structural changes and defective recognition functions. Journal of the American Society of Nephrology: JASN, 17(1), 170–177. https://doi.org/10.1681/ASN.2005080868

Klein, R. J., Zeiss, C., Chew, E. Y., Tsai, J. Y., Sackler, R. S., Haynes, C., Henning, A. K., SanGiovanni, J. P., Mane, S. M., Mayne, S. T., Bracken, M. B., Ferris, F. L., Ott, J., Barnstable, C., & Hoh, J. (2005). Complement factor H polymorphism in age-related macular degeneration. Science, 308(5720), 385–389. https://doi.org/10.1126/science.1109557

Laine, M., Jarva, H., Seitsonen, S., Haapasalo, K., Lehtinen, M. J., Lindeman, N., Anderson, D. H., Johnson, P. T., Järvelä, I., Jokiranta, T. S., Hageman, G. S., Immonen, I., & Meri, S. (2007). Y402H polymorphism of complement factor H affects binding affinity to C-reactive protein. Journal of Immunology (Baltimore, Md. : 1950), 178(6), 3831–3836. https://doi.org/10.4049/jimmunol.178.6.3831

Le Quintrec, M., Lionet, A., Kamar, N., Karras, A., Barbier, S., Buchler, M., Fakhouri, F., Provost, F., Fridman, W. H., Thervet, E., Legendre, C., Zuber, J., & Frémeaux-Bacchi, V. (2008). Complement mutation-associated de novo thrombotic microangiopathy following kidney transplantation. American journal of transplantation : official journal of the American Society of Transplantation and the American Society of Transplant Surgeons, 8(8), 1694–1701. https://doi.org/10.1111/j.1600-6143.2008.02297.x

Loeven, M. A., Rops, A. L., Lehtinen, M. J., van Kuppevelt, T. H., Daha, M. R., Smith, R. J., Bakker, M., Berden, J. H., Rabelink, T. J., Jokiranta, T. S., & van der Vlag, J. (2016). Mutations in Complement Factor H Impair Alternative Pathway Regulation on Mouse Glomerular Endothelial Cells in Vitro. The Journal of biological chemistry, 291(10), 4974–4981. https://doi.org/10.1074/jbc.M115.702506

Malik, T. H., Lavin, P. J., Goicoechea de Jorge, E., Vernon, K. A., Rose, K. L., Patel, M. P., de Leeuw, M., Neary, J. J., Conlon, P. J., Winn, M. P., & Pickering, M. C. (2012). A hybrid CFHR3-1 gene causes familial C3 glomerulopathy. Journal of the American Society of Nephrology : JASN, 23(7), 1155–1160. https://doi.org/10.1681/ASN.2012020166

Martin Merinero, H., Subías, M., Pereda, A., Gómez-Rubio, E., Juana Lopez, L., Fernandez, C., Goicoechea de Jorge, E., Martin-Santamaria, S., Cañada, F. J., & Rodríguez de Córdoba, S. (2021). Molecular bases for the association of FHR-1 with atypical hemolytic uremic syndrome and other diseases. Blood, 137(25), 3484–3494. https://doi.org/10.1182/blood.2020010069

Medjeral-Thomas, N., Malik, T. H., Patel, M. P., Toth, T., Cook, H. T., Tomson, C., & Pickering, M. C. (2014). A novel CFHR5 fusion protein causes C3 glomerulopathy in a family without Cypriot ancestry. Kidney international, 85(4), 933–937. https://doi.org/10.1038/ki.2013.348

Medjeral-Thomas, N. R., Lomax-Browne, H. J., Beckwith, H., Willicombe, M., McLean, A. G., Brookes, P., Pusey, C. D., Falchi, M., Cook, H. T., & Pickering, M. C. (2017). Circulating complement factor H–related proteins 1 and 5 correlate with disease activity in IgA nephropathy. Kidney International, 92(4), 942–952. https://doi.org/10.1016/j.kint.2017.03.043

Merinero, H. M., García, S. P., García-Fernández, J., Arjona, E., Tortajada, A., & Rodríguez de Córdoba, S. (2018). Complete functional characterization of disease-associated genetic variants in the complement factor H gene. Kidney international, 93(2), 470–481. https://doi.org/10.1016/j.kint.2017.07.015

Mohlin, F. C., Nilsson, S. C., Levart, T. K., Golubovic, E., Rusai, K., Müller-Sacherer, T., Arbeiter, K., Pállinger, É., Szarvas, N., Csuka, D., Szilágyi, Á., Villoutreix, B. O., Prohászka, Z., & Blom, A. M. (2015). Functional characterization of two novel non-synonymous alterations in CD46 and a Q950H change in factor H found in atypical hemolytic uremic syndrome patients. Molecular immunology, 65(2), 367–376. https://doi.org/10.1016/j.molimm.2015.02.013

Montes, T., Goicoechea de Jorge, E., Ramos, R., Gomà, M., Pujol, O., Sánchez-Corral, P., & Rodríguez de Córdoba, S. (2008). Genetic deficiency of complement factor H in a patient with age-related macular degeneration and membranoproliferative glomerulonephritis. Molecular Immunology, 45(10), 2897–2904. https://doi.org/10.1016/j.molimm.2008.01.027

Neumann, H. P., Salzmann, M., Bohnert-Iwan, B., Mannuelian, T., Skerka, C., Lenk, D., Bender, B. U., Cybulla, M., Riegler, P., Königsrainer, A., Neyer, U., Bock, A., Widmer, U., Male, D. A., Franke, G., & Zipfel, P. F. (2003). Haemolytic uraemic syndrome and mutations of the factor H gene: a registry-based study of German speaking countries. Journal of medical genetics, 40(9), 676–681. https://doi.org/10.1136/jmg.40.9.676

Noris, M., Caprioli, J., Bresin, E., Mossali, C., Pianetti, G., Gamba, S., Daina, E., Fenili, C., Castelletti, F., Sorosina, A., Piras, R., Donadelli, R., Maranta, R., van der Meer, I., Conway, E. M., Zipfel, P. F., Goodship, T. H., & Remuzzi, G. (2010). Relative role of genetic complement abnormalities in sporadic and familial aHUS and their impact on clinical phenotype. Clinical journal of the American Society of Nephrology : CJASN, 5(10), 1844–1859. https://doi.org/10.2215/CJN.02210310

Pechtl, I. C., Kavanagh, D., McIntosh, N., Harris, C. L., & Barlow, P. N. (2011). Disease-associated N-terminal complement factor H mutations perturb cofactor and decay-accelerating activities. The Journal of biological chemistry, 286(13), 11082–11090. https://doi.org/10.1074/jbc.M110.211839

Recalde, S., Tortajada, A., Subias, M., Anter, J., Blasco, M., Maranta, R., Coco, R., Pinto, S., Noris, M., García-Layana, A., & Rodríguez de Córdoba, S. (2016). Molecular Basis of Factor H R1210C Association with Ocular and Renal Diseases. Journal of the American Society of Nephrology : JASN, 27(5), 1305–1311. https://doi.org/10.1681/ASN.2015050580

Sánchez-Corral, P., Pérez-Caballero, D., Huarte, O., Simckes, A. M., Goicoechea, E., López-Trascasa, M., & de Córdoba, S. R. (2002). Structural and functional characterization of factor H mutations associated with atypical hemolytic uremic syndrome. American journal of human genetics, 71(6), 1285–1295. https://doi.org/10.1086/344515

Servais, A., Noël, L.-H., Roumenina, L. T., le Quintrec, M., Ngo, S., Dragon-Durey, M.-A., Macher, M.-A., Zuber, J., Karras, A., Provot, F., Moulin, B., Grünfeld, J.-P., Niaudet, P., Lesavre, P., & Frémeaux-Bacchi, V. (2012). Acquired and genetic complement abnormalities play a critical role in dense deposit disease and other C3 glomerulopathies. Kidney International, 82(4), 454–464. https://doi.org/10.1038/ki.2012.63

Szarvas, N., Szilágyi, Á., Csuka, D., Takács, B., Rusai, K., Müller, T., Arbeiter, K., Réti, M., Haris, Á., Wagner, L., Török, S., Kelen, K., Szabó, A. J., Reusz, G. S., Morgan, B. P., & Prohászka, Z. (2016). Genetic analysis and functional characterization of novel mutations in a series of patients with atypical hemolytic uremic syndrome. Molecular immunology, 71, 10–22. https://doi.org/10.1016/j.molimm.2016.01.003

Tortajada, A., Yébenes, H., Abarrategui-Garrido, C., Anter, J., García-Fernández, J. M., Martínez-Barricarte, R., Alba-Domínguez, M., Malik, T. H., Bedoya, R., Cabrera Pérez, R., López Trascasa, M., Pickering, M. C., Harris, C. L., Sánchez-Corral, P., Llorca, O., & Rodríguez de Córdoba, S. (2013). C3 glomerulopathy-associated CFHR1 mutation alters FHR oligomerization and complement regulation. The Journal of clinical investigation, 123(6), 2434–2446. https://doi.org/10.1172/JCI68280

Tortajada, A., Gutiérrez, E., Goicoechea de Jorge, E., Anter, J., Segarra, A., Espinosa, M., Blasco, M., Roman, E., Marco, H., Quintana, L. F., Gutiérrez, J., Pinto, S., Lopez-Trascasa, M., Praga, M., & Rodriguez de Córdoba, S. (2017). Elevated factor H-related protein 1 and factor H pathogenic variants decrease complement regulation in IgA nephropathy. Kidney international, 92(4), 953–963. https://doi.org/10.1016/j.kint.2017.03.041

Venables, J. P., Strain, L., Routledge, D., Bourn, D., Powell, H. M., Warwicker, P., Diaz-Torres, M. L., Sampson, A., Mead, P., Webb, M., Pirson, Y., Jackson, M. S., Hughes, A., Wood, K. M., Goodship, J. A., & Goodship, T. H. J. (2006). Atypical haemolytic uraemic syndrome associated with a hybrid complement gene. PLoS Medicine, 3(10), e431. https://doi.org/10.1371/journal.pmed.0030431

Xiao, X., Ghossein, C., Tortajada, A., Zhang, Y., Meyer, N., Jones, M., Borsa, N. G., Nester, C. M., Thomas, C. P., de Córdoba, S. R., & Smith, R. J. (2016). Familial C3 glomerulonephritis caused by a novel CFHR5-CFHR2 fusion gene. Molecular immunology, 77, 89–96. https://doi.org/10.1016/j.molimm.2016.07.007

Xu, B., Kang, Y., Du, Y., Guo, W., Zhu, L., & Zhang, H. (2022). Atypical Hemolytic Uremic Syndrome-Associated FHR1 Isoform FHR1*B Enhances Complement Activation and Inflammation. Frontiers in immunology, 13, 755694. https://doi.org/10.3389/fimmu.2022.755694

Yu, Y., Triebwasser, M. P., Wong, E. K., Schramm, E. C., Thomas, B., Reynolds, R., Mardis, E. R., Atkinson, J. P., Daly, M., Raychaudhuri, S., Kavanagh, D., & Seddon, J. M. (2014). Whole-exome sequencing identifies rare, functional CFH variants in families with macular degeneration. Human molecular genetics, 23(19), 5283–5293. https://doi.org/10.1093/hmg/ddu226

Wagner, E. K., Raychaudhuri, S., Villalonga, M. B., Java, A., Triebwasser, M. P., Daly, M. J., Atkinson, J. P., & Seddon, J. M. (2016). Mapping rare, deleterious mutations in Factor H: Association with early onset, drusen burden, and lower antigenic levels in familial AMD. Scientific reports, 6, 31531. https://doi.org/10.1038/srep31531

Wong, E. K. S., Hallam, T. M., Brocklebank, V., Walsh, P. R., Smith-Jackson, K., Shuttleworth, V. G., Cox, T. E., Anderson, H. E., Barlow, P. N., Marchbank, K. J., Harris, C. L., & Kavanagh, D. (2020). Functional Characterization of Rare Genetic Variants in the N-Terminus of Complement Factor H in aHUS, C3G, and AMD. Frontiers in Immunology, 11, 602284. https://doi.org/10.3389/fimmu.2020.602284

Zareparsi, S., Branham, K. E. H., Li, M., Shah, S., Klein, R. J., Ott, J., Hoh, J., Abecasis, G. R., & Swaroop, A. (2005). Strong association of the Y402H variant in complement factor H at 1q32 with susceptibility to age-related macular degeneration. American Journal of Human Genetics, 77(1), 149–153. https://doi.org/10.1086/431426

Zhai, Y. L., Meng, S. J., Zhu, L., Shi, S. F., Wang, S. X., Liu, L. J., Lv, J. C., Yu, F., Zhao, M. H., & Zhang, H. (2016). Rare Variants in the Complement Factor H-Related Protein 5 Gene Contribute to Genetic Susceptibility to IgA Nephropathy. Journal of the American Society of Nephrology : JASN, 27(9), 2894–2905. https://doi.org/10.1681/ASN.2015010012
